# Genome Physical Mapping of Polyploids: A BIBAC Physical Map of Cultivated Tetraploid Cotton, *Gossypium hirsutum* L

**DOI:** 10.1371/journal.pone.0033644

**Published:** 2012-03-16

**Authors:** Meiping Zhang, Yang Zhang, James J. Huang, Xiaojun Zhang, Mi-Kyung Lee, David M. Stelly, Hong-Bin Zhang

**Affiliations:** 1 Department of Soil and Crop Sciences, Texas A&M University, College Station, Texas, United States of America; 2 College of Life Science, Jilin Agricultural University, Changchun, Jilin, China; Kyushu Institute of Technology, Japan

## Abstract

Polyploids account for approximately 70% of flowering plants, including many field, horticulture and forage crops. Cottons are a world-leading fiber and important oilseed crop, and a model species for study of plant polyploidization, cellulose biosynthesis and cell wall biogenesis. This study has addressed the concerns of physical mapping of polyploids with BACs and/or BIBACs by constructing a physical map of the tetraploid cotton, *Gossypium hirsutum* L. The physical map consists of 3,450 BIBAC contigs with an N50 contig size of 863 kb, collectively spanning 2,244 Mb. We sorted the map contigs according to their origin of subgenome, showing that we assembled physical maps for the A- and D-subgenomes of the tetraploid cotton, separately. We also identified the BIBACs in the map minimal tilling path, which consists of 15,277 clones. Moreover, we have marked the physical map with nearly 10,000 BIBAC ends (BESs), making one BES in approximately 250 kb. This physical map provides a line of evidence and a strategy for physical mapping of polyploids, and a platform for advanced research of the tetraploid cotton genome, particularly fine mapping and cloning the cotton agronomic genes and QTLs, and sequencing and assembling the cotton genome using the modern next-generation sequencing technology.

## Introduction

Polyploidy is a significant evolutionary process in higher organisms. It has long been recognized as a prominent speciation process in plants as well as some fishes [1], [2]. The genomes of most angiosperms are thought to have incurred one or more polyploidization events during evolution [3]. Studies have demonstrated that genome doubling has also been significant in the evolutionary history of all vertebrates and in many other eukaryotes [4]–[7]. It is estimated that about 70% of the extant angiosperms are polyploids, including many world-leading field, forage, horticultural and environmental crops such as cotton, wheat, potatoes, canola, sugarcane, oats, peanut, tobacco, rose, alfalfa, coffee and banana. Nevertheless, genomics research of polyploids is generally behind that of diploid species due to their polyploidy nature that could significantly complicate genome research, especially genome physical mapping with large-insert bacterial artificial chromosome (BAC) and/or transformation-competent binary BAC (BIBAC) clones. BAC and/or BIBAC-based genome physical maps have been demonstrated to be the centerpiece essential for many areas of advanced studies such as gene and quantitative trait locus (QTL) fine mapping and cloning, genome sequencing, functional genomics, and comparative genomics. Therefore, genome-wide physical maps have been developed from BACs and/or BIBACs for a number of diploid species [8]–[23]. However, no physical map has been developed and no genome sequenced to date for a polyploid species though the feasibility of constructing a physical map of a polyploidy plant species by BAC fingerprint analysis was tested using an *in silico* merged BAC library of two wheat homoeologous arms, 3AS and 3DS [24]. This study has addressed the concerns of genome physical mapping of polyploids with BACs and/or BIBACs using Upland cotton, *Gossypium hirsutum* L.

Upland cotton is an allotetraploid, consisting of A- and D-subgenomes, and has a genome size of approximately 2,400 Mb/1C [25]. It was originated around 1–2 million years ago via allopolyploidization between a diploid species containing an A genome such as *G. herbaceum* (A_1_) or *G. arboreum* (A_2_) and a diploid species containing a D genome such as *G. raimondii* (D_5_) or *G. gossypioides* (D_6_), whereas the A- and D-subgenomes are homoeologous [26], their diploid progenitors having splided from a common ancestor some 5–7 million years ago [27]–[31].

Cottons are a world leading fiber and oilseed crop, the textile and bioenergy industries feed-stocked by cotton fibers and oilseeds perhaps contributing thousands of billion dollars to the world's economy. Upland cotton economically is the most important among the four cultivated cotton species, *G. hirsutum* (AD_1_), *G. barbadense* (AD_2_), *G. herbaceum* (A_1_) and *G. arboreum* (A_2_), providing over 90% of the world's cotton fibers and oilseeds. Furthermore, since the cotton polyploid complex consists of extant allotetraploids (including Upland cotton) and diploid relatives (for review, see [32]), it has long been used as a model species for studies of plant polyploidization, speciation and evolution. Finally and importantly, cotton fibers are a model system for studies of cellulose biosynthesis that is crucial to bioenergy production and plant cell wall biogenesis that makes the largest portion of biomass on the earth. This is because cotton fibers are originated from single individual cells and approximately 90% of their makeup is celluloses that are the largest component of plant cell walls. Therefore, cotton genomics research is of significance in numerous aspects economically and scientifically.

Cotton genome research has been pursued extensively in the past 20 years. A large number of DNA markers and several genetic maps have been constructed, hundreds of QTLs important to fiber yield and quality mapped, a large collection of expressed sequence tags (ESTs) generated and several large-insert BAC and BIBAC libraries developed for cotton (for review, see ref. 32). Recently, a draft physical map has been developed from BACs [33] and whole-genome draft sequences generated for a wild diploid relative of the Upland cotton D-subgenome, *G. raimondii* (D_5_) (http://www.ncbi.nlm.nih.gov/sra/SRA024364?report=full). Nevertheless, the D genome of the wild species was too diverged to be claimed as the diploid donor of the Upland cotton D-subgenome [34] and could not be used to study the molecular basis of the economically important cotton fiber yield and quality. That the genome of the wild species was physically mapped and sequenced was mainly due to the concern about the genome complexity of the cultivated tetraploid cottons (*G. hirsutum* and *G. barbadense*). Neither whole genome physical map nor whole genome sequence has been reported to date for any of the polyploid cottons, including Upland cotton.

In this study, we have addressed the feasibility and developed a strategy of constructing whole genome physical maps of polyploid species from large-insert BACs and/or BIBACs by capillary electrophoresis-based fingerprint analysis. We constructed a physical map of the tetraploid Upland cotton from a large-insert BIBAC library and sorted the physical map contigs according to their origin of A- or D-subgenome, thus showing that the physical maps for the A- and D-subgenomes of Upland cotton have been constructed, separately. We have identified the BIBAC contigs containing the loci of genes important to fiber development, fiber cellulose biosynthesis, cell wall biogenesis, seed fatty acid metabolism, and cotton-nematode interaction. Furthermore, to facilitate the use of the physical map for sequencing the Upland cotton genome using the modern next-gen sequencing technology, we have tagged the physical map using nearly 10,000 BIBAC end sequences (BESs), with one BES-STS (sequence-tagged site) in approximately every 250 kb, and identified the minimal tilling path (MTP) clones of the physical map. These results provide a line of evidence and a strategy for genome physical mapping of polyploids with BACs and/or BIBACs and a framework essential for sequencing the tetraploid Upland cotton genome using the modern next-gen sequencing technology and many other advanced studies, such as plant polyploidization, speciation and evolution, plant cellulose biosynthesis, plant cell wall biogenesis, and fine mapping and cloning of genes and QTLs of agronomic importance. Additionally, the use of the plant-transformation-competent BIBAC library in the Upland cotton physical map will further facilitate large-scale functional analysis of the Upland cotton genome and gene/QTL cloning in the post-genome era.

## Results

### Upland cotton BIBAC physical map

A total of 76,800 BIBAC clones [35] were fingerprinted for Upland cotton physical map assembly and the feasibility test of genome physical mapping of polyploids by fingerprint analysis (**Table 1**). Of the fingerprints, 73,983 (96.3%) passed our fingerprint quality check (MATERIALS AND METHODS) and thus were validated for physical map assembly. This number of clones corresponds to approximately 4.2-fold coverage of the haploid *G. hirsutum* genome if it has a genome size of 2,400 Mb/1C. The BIBAC clones had an average number of 38.3 restriction fragment bands per clone in the window of 35–500 bases.

**Table 1:** Summary of the allotetraploid Upland cotton genome physical map.

Clones fingerprinted	76,800 (4.3×)
Clones used in the physical map construction	73,983 (4.2×)
Singletons	9,963
Clones contained in the contigs	64,020
Contigs assembled	3,450
Contigs containing >100 clones	39
Contigs containing 50–99 clones	198
Contigs containing 25–49 clones	509
Contigs containing 10–24 clones	1,018
Contigs containing 4–9 clones	1,273
Contigs containing 3 clones	413
Average contig size	650 kb
N50 contig size	863 kb
Largest contig size	6,380 kb
Average clone number per contig	19
Average band number per clone	38.3
Physical length contribution per clone	35 kb
BESs contained in the contigs	9,711
Consensus bands of the contigs	636,530
Total physical length of the physical map	2,244 Mb
Clones in the physical map MTP	15,277
Total physical length of the MTP clones	1,955 Mb

Automatic contigs were assembled from the validated BIBAC fingerprints, edited, verified and extended. We first manually checked every contig for potential chimeric contigs based on its BIBAC fingerprint patterns. All questionable contigs were split and re-assembled at a higher stringency using cutoff values ranging from 1e-06 to 1e-10. Then, we identified the potential junctions between contigs and merged them into larger ones. The entire fingerprint database was searched for matches to the fingerprints of the terminal clone of every contig using the End to End Function of the FingerPrinted Contig (FPC) program with cutoff values ranging from 1e-20 to 1e-06. Contigs were merged only if their terminal clones shared 5 or more bands and their overall fingerprint patterns supported the mergence. Finally, singletons were added to the contigs, without any further mergence, if they were overlapped with one or more clones in a contig using cutoff values between 1e-20 and 1e-04. This map assembly strategy resulted in a genome-wide physical map of 3,450 BIBAC contigs. Of the 73,983 BIBAC clones used in the physical map assembly, 64,020 were assembled into contigs, whereas the remaining 9,963 clones remained as singletons.

**Figure 1:**
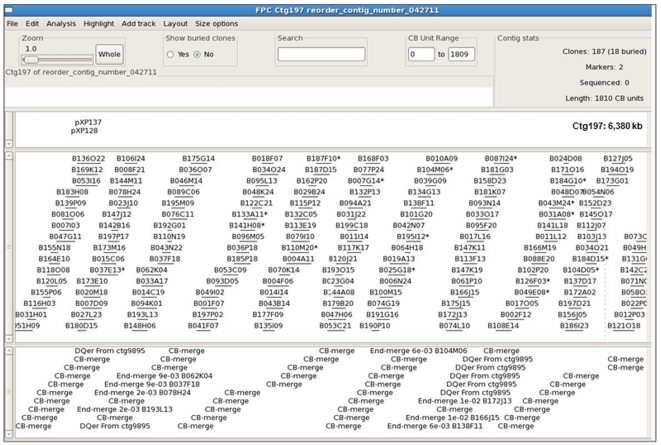
An A-subgenome BIBAC contig of the Upland cotton physical map. The contig consists of 187 BIBACs, spanning 6,380 kb in physical length or approximately 13 cM in genetic distance.

**Table 1** summarizes the characteristics of the tetraploid Upland cotton BIBAC contig map (for all contigs of the physical map, see **Table S2**). Each contig contains 3 to 195 BIBAC clones, with an average number of 19 clones per contig. The contigs have a physical length ranging from 84 to 6,380 kb, with an average physical length of 650 kb and an N50 contig size of 863 kb (**Fig. 1**). The 3,450 contigs of the physical map consists of 636,530 unique consensus bands (CBs), with each clone contributing an average of 9.9 unique CBs or approximately 35.0 kb to the physical map. It was estimated that the contigs collectively span approximately 2,244 Mb, accounting for 92.6% of the Upland cotton TM-1 genome [25]. Moreover, the 9,711 BESs of the BIBACs sequenced previously [35] were incorporated into the cotton physical map manually, thus making one BES-STS in approximately 250 kb along the cotton genome physical map.

### Evaluation of physical map contigs and identification of the contigs containing genes of interest

Two approaches were used to evaluate the reliability of the cotton physical map contigs. In the first approach, we assembled contigs from the BIBAC fingerprints using two different contig building strategies independently by different scientists and then compared the resultant contigs, as described by Wu *et al.* [16]. The result showed that 99.6% of the contigs resulted from the two strategies was completely consistent in both clone content and order, suggesting that the two sets of contigs were assembled properly. In the second approach, we screened the source BIBAC library of the physical map with 13 single- or low-copy overgo probes designed from the unique regions of genes important to cotton fiber development (*MYBB*, *MYBT2*, *RDL1*), fiber cellulose biosynthesis (*CelA1*, *CelA3*, *CelA6*, *GhCesA2*, *GhIRX3*, *GhCesA3*, a unnamed *GhCes*), seed fatty acid metabolism (*FADO6*) and cotton-nematode interaction (*MIC3*, *MIC1-15*). With each of the overgo probes, from 2 to 8 positive clones were identified (**Table 2**). The positive clones of each of *CelA1*, *RDL1*, *FADO6*, *MYBB*, *GhCesA2*, *GhRx3* and *GhCesA3* were located to a single contig, and the positive clones of each of *CelA3*, *CelA6*, *MYBT2* and *GhCes* were located to two contigs. Together, the positive clones of each of 11 (84.6%) of the 13 gene overgos were located to a single or two contigs. Since the physical map source BIBAC library was constructed from the tetraploid Upland cotton containing two homoeologous subgenomes, it is expected that the positive clones of a single-locus probe in the two or one subgenome are assembled into one or two contigs. Therefore, we concluded that the physical map contigs were assembled properly (for additional evidence of the map quality, also see below). The remaining two gene overgo probes, *MIC-3* and *MIC1-15*, which belong to the *Meloidogyne*-induced protein gene family, hybridized to the same set of BIBACs; their positive clones were thus identified in the same set of contigs including Ctg1816, Ctg2583, Ctg2716 and Ctg2723, suggesting that two genes are physically closed to each other.

**Table 2:** Identification of contigs containing genes of interest and verification of the physical map.

Gene	GenBank acc. No.	Annotation	Positive clones*	Contig identified	Sub-genome assignment
*CelA1*	HQ143024.1	Cellulose synthase A1	**B130C07, B016N19**	Ctg1108 (556 kb)	Unassigned
*CelA3*	HQ143030.1	Cellulose synthase A3	**B024E21, B072G17, B086A07, B083I01, B073K01, B073L02, B096F08**	Ctg979 (884 kb) Ctg2187 (1,212 kb)	Unassigned A
*CelA6*	GQ200733.1	Cellulose synthase catalytic subunit A3	B098I23, **B116B12, B017I05**, B035E07, **B115K16**, B005M05, **B086H21**	Ctg364 (1,367 kb) Ctg2189 (800 kb)	A A
*MIC3*	GQ231916.1	*Meloidogyne*-induced cotton protein 3	**B099A19, B024H22, B014D05, B027E13, B026F22, B092E14**	Ctg1816 (299 kb) Ctg2583 (225 kb) Ctg2716 (271 kb) Ctg2723 (874 kb)	Unassigned A Unassigned A
	EU025993	*Meloidogyne*-induced cotton protein 1–15	**B099A19, B024H22, B014D05, B027E13, B026F22, B092E14**	Ctg1816 (299 kb) Ctg2583 (225 kb) Ctg2716 (271 kb) Ctg2723 (874 kb)	Unassigned A Unassigned A
*RDL1*	AY633558.1	GaRDL1 gene, promoter region	B175F03, B050B20, **B187C03, B186M07, B016L07, B166C05, B161G09**	Ctg1397 (831 kb)	A
*FADO6*	Y10112.2	Fatty acid desaturase omega-6	**B138P05, B080A19**	Ctg1156 (2,079 kb)	A
*MYBB*	AF034130.1	MYB-like DNA-binding domain protein	**B174C01, B192C23**	Ctg1137 (768 kb)	Unassigned
*MYBT2*	AY366352.1	MYB-like transcription factor 2	**B026F22, B007F03**	Ctg2723 (874 kb) Ctg3247 (831 kb)	A A
*GhCesA2*	U58284.1	Secondary wall cellulose synthase A2	**B048N17, B085P21, B162F12**, B046G15	Ctg3423 (1,614 kb)	A, D
*GhIRX3*	DT048689	Irregular xylem 3/cellulose synthase A7	**B070C20**, B173F02	Ctg1090 (2,671 kb)	D
*GhCesA3*	AF150630.2	Primary wall cellulose synthase	**B075L23, B097O20, B108L10, B146H15, B178M15**, B170C06, B009G10, B065P05	Ctg258 (331 kb)	Unassigned
*GhCes*	AF150630	Unknown cellulose synthase	**B145L18 B164E04, B165A23**, B008D22	Ctg258 (331 kb) Ctg2187 (1,212 kb)	Unassigned A

* The positive clones of the genes were identified previously by screening the physical map source BIBAC library using overgo probes designed from the gene sequences [34]. The clones not bolded in the table indicate singletons.

### Identification of A- and D-subgenome physical maps

To test whether the physical map contigs were A- or D-subgenome-specific, as is their origin of subgenome, or assembled in a mix of A- and D-subgenome clones, we further screened the source BIBAC library with the probes derived from three A- or D-subgenome-specific, interspersed repetitive elements, pXP128, pXP137 and pXP195 [36], [37], respectively. Of the three repetitive elements, pXP128 and pXP137 were A-subgenome-specific and pXP195 was D-subgenome-specific [36], [37]. Therefore, the positive clones identified with the probes pXP128 and p XP137 are expected to be A-subgenome-originated whereas those identified with the probe pXP195 are expected to be D-subgenome-originated. The library hybridization resulted in 2,056, 1,148 and 523 positive BIBAC assignments for probes pXP128, pXP137 and pXP195, respectively (for the list of the positive clones of pXP128, pXP137 and pXP195, see **Table S3**). These positive clones were incorporated into a total of 1,211 (34.9%) of the 3,450 physical map contigs. Of these 1,211 contigs, 1,063 collectively spanning 1,095 Mb of the total physical length all contained the positive clones identified by the A-subgenome-specific probes (pXP128 and pXP135), suggesting that the contigs were originated from A-subgenome. One hundred forty-one of the 1,211 contigs accounting for 97 Mb of the total physical length all contained the positive clones identified by the D-subgenome-specific probe (pXP195), suggesting that the contigs were originated from D-subgenome. The remaining seven contigs, only accounting for 0.58% of the 1,211 contigs and collectively spanning approximately 5.98 Mb, contained the mixed positive clones identified by the A- and D-subgenome-specific probes (for a detailed list of the subgenome-specific contigs, see **Table S2**). These results suggest that the physical map contigs were assembled according to their origin of A- or D-subgenome; thus, A- and D-subgenome physical maps have been developed, separately. These results also provided an additional line of evidence on the reliability of the physical map assembly because a much larger number of the contigs would contain the positive clones identified by the three probes if they were assembled improperly.

### Identification of MTP clones of the physical map

The MTP clones of a physical map have been widely used to sequence a complex genome using either the Sanger method or the next-gen sequencing technology. Since physical maps developed for the A- and D-subgenomes of Upland cotton in this study provide a platform for sequencing the tetraploid Upland cotton genome using the next-gen sequencing technology, we identified the MTP clones of the Upland cotton physical map. We identified a total of 15,277 MTP clones of the cotton physical map (**Table 1**). These MTP clones collectively spanned 1,955 Mb, approximately 81.5% of the Upland cotton genome.

## Discussion

We have developed a genome-wide BIBAC-based physical map of the tetraploid Upland cotton, *G. hirsutum*, cv. TM-1. The physical map consists of 3,450 overlapping BIBAC contigs. Each contig contains 3 to 195 clones, with an average number of 19 clones per contig. Each clone contributes an average of 9.9 unique CBs or approximately 35.0 kb to the physical length of the contig assembly. The contigs have physical sizes ranging from 84 to 6,380 kb, with an average physical length of 650 kb and an N50 contig size of 863 kb. The 3,450 contigs constituting the Upland cotton physical map together consist of 636,530 unique CBs spanning a total length of approximately 2,244 Mb, equivalent about 92.6% of the estimated Upland cotton TM-1 genome [25]. We have verified the contigs constituting the Upland cotton physical map using two different approaches. The results from both approaches indicate that the physical map contigs have been assembled properly. Moreover, this conclusion has been also further confirmed by the result that over 99% of the positive clones identified by A- or D-subgenome-specific probes were assembled into contigs according to their origin of subgenome, separately, with few contigs containing both A- and D-subgenome specific positive clones. Furthermore, we have demonstrated that the physical map contigs are assembled according to their origin of A- or D-subgenome and identified 1,063 A-subgenome-specific contigs and 141 D-subgenome specific contigs, together spanning 1,192 Mb accounting for 50% of the Upland cotton genome. Finally, we have integrated approximately 10,000 BESs, with an average of 2.8 BES-STS per contig, into the contig map; hence, the map has been marked, with one BES-STS in approximately every 250 kb. In addition, since the physical map is constructed from a BIBAC library that is competent to be transformed into plants directly either via *Agrobatcerium* [38], [39] or by biolistic bombardment [40], [41], it could be directly used for large-scale functional analysis of the Upland cotton genome, gene/QTL cloning and molecular breeding. Therefore, the physical map provides a platform and tools necessary for many areas of advanced research, particularly gene/QTL fine mapping and cloning, large-scale genome sequencing and large-scale functional analysis of the Upland cotton genome.

Polyploids account for approximately 70% of the flowering plant species. Of the species, many are economically important, but the physical maps of few have been developed due to the nature of their polyploidy, even though physical maps have been proven to be the centerpiece for many advanced genomics studies. This study, with that of allopolyploid wheat homoeologous chromosomal arms [24], has demonstrated that it is feasible to develop the genome physical maps of polyploids from large-insert BACs and/or BIBACs by fingerprint analysis, with a vast majority of the contigs assembled according to their origin of subgenome, and provided a strategy of sorting the physical map contigs according to their origin of subgenome using subgenome-specific, interspersed repeated sequences. We have identified a total of 1,211 contigs from the physical map using three subgenome-specific, interspersed repeated sequence probes. Out of the 1,211 contigs, 1,204 (99.4%) contain A- or D-subgenome-specific positive clones; only seven (0.6%) containing mixed A- and D-subgenome-specific positive clones (**Table S3**). This result suggests that the widely-used BAC or BIBAC fingerprint analysis and contig assembly method is suitable for whole-genome physical mapping of polyploids, at least for some of them, if not all. The 1,204 contigs account for 34.9% of the 3,450 contigs constituting the Upland cotton physical map; additional subgenome-specific, interspersed repeated sequence probes will be needed to sort the remaining contigs of the physical map. Alternatively, they could be sorted according to their origin of subgenome while the physical map is integrated with a cotton genetic map.

The Upland cotton physical map provides a powerful platform essential for many aspects of advanced research of the Upland cotton and other *Gossypium* genomes. We have identified the MTP clones of the physical map according to their fingerprints and overlaps with neighboring clones. These MTP clones, along with the 10,000 BESs integrated into the physical map, make it possible to properly sequence and assemble the tetraploid Upland cotton genome using the next-gen sequencing technology. Furthermore, the selection of the MTP clones has further eliminated the questionable-clones (Q-clones), if any, from the contigs to be sequenced. Sequencing the Upland cotton genome will significantly promote cotton genomics research because the availability of whole genome quality sequences has been proven to be crucial to many advanced molecular studies and approximately 90% of the world's cotton is produced by the cotton species. We have also identified contigs from the physical maps that contain the loci of a number of genes of agronomic importance, such as those involved in cotton fiber development, cellulose biosynthesis, cell wall biogenesis, seed fatty acid metabolism and cotton-nematode interaction. These contigs have provided tools essential for detailed characterization of the genes at the genomic level and comparative analysis of their loci between A- and D-subgenomes and between the subgenomes of the tetraploid cotton and the genomes of their diploid relatives, thus addressing the formation and evolution of polyploid cottons.

The Upland cotton physical map reported in this study represents the first physical map of the tetraploid Upland cotton. Efforts will be needed to further improve the genome coverage of the physical map, to integrate the physical map with the developed genetic maps of the species and to sort all contigs of the physical map according to their origin of subgenome. These experiments could be done by analyzing additional large-insert BACs and/or BIBACs, and developing and integrating with a high-density SNP map of the Upland cotton genome using the next-gen sequencing technology. This process could be readily pursued concurrently with the Upland cotton genome sequencing using the physical map. Nevertheless, the separate assembly of A- and D-subgenome-specific contig maps in this study has already shed light on sequencing and assembling the tetraploid Upland cotton genome with a physical map such as this one as a platform using the next-gen sequencing technology.

## Materials and Methods

### Source BIBAC library

A BIBAC library constructed from *G. hirsutum* cv. Texas Marker-1 (TM-1) [35] was used in this study. The BIBAC library was constructed from the cotton nuclear DNA partially digested with *Bam*H I in a BIBAC vector pCLD04541. It contains 76,800 clones and has an average insert size of 135 kb, thus providing a 4.3-fold coverage of the haploid Upland cotton genome. Additionally, since the library was constructed in the BIBAC vector, its clones can be transformed into plants directly either via *Agrobacterium* [38], [39] or by biolistic bombardment [40], [41], thus well-suited for large-scale functional analysis of the cotton genome and gene/QTL cloning. The library is available to the public through the GENE*finder* Genomic Resources at Texas A&M University, College Station, Texas, USA.

### BIBAC DNA isolation and fingerprinting

Two methods have been previously developed and used to generate fingerprints from large-insert BACs and/or BIBACs for genome physical mapping by capillary electrophoresis-based fingerprint analysis, including the one fluorescent dye-labeling method and multiple fluorescent dye-labeling SNaPshot method [22], [42]. The one fluorescent dye-labeling method digests a BAC or BIBAC DNA with 2–4 restriction enzymes, depending on the number of bands desirable for physical map assembly, and end-labels the restricted fragments with one fluorescent dye [22], [42]. Therefore, this method can be multiplexed readily by labeling different BACs or BIBACs with different fluorescent dyes. The multiple fluorescent dye-labeling SNaPshot method digests a BAC or BIBAC DNA with five restriction enzymes and end-labels the restricted fragments with four fluorescent dyes [42]; thus, it is difficult to be multiplexed. Recent physical mapping studies showed that the one fluorescent dye-labeling method is not only several-fold more economic, but also often generates much higher-quality physical maps, including larger contigs and fewer questionable (Q) clones [17], [18], [21], [22], [43], [44], than the multiple fluorescent dye-labeling method [45]–[50]. Therefore, we used the one fluorescent dye-labeling method in this study to construct the physical map of the tetraploid Upland cotton genome.

Our previous studies demonstrated that BAC/BIBAC fingerprints generated with different restriction enzyme combinations resulted in physical maps of different qualities [17], [44]. Therefore, we first tested twenty-four 3-, 4- and 5-enzyme combinations of *Bam*H I, *Eco*R I, *Hin*d III, *Xba* I, *Xho* I, and *Hae* III on 96 BIBACs randomly selected from the Upland cotton BIBAC library. Only the fragment ends produced by *Bam*H I, *Eco*R I, *Hin*d III, *Xba* I or *Xho* I digestion were labeled using NED-ddATP or HEX-ddATP (see below). The restriction enzyme *Hae* III is used to digest the labeled fragments into sizes that allow separation on a capillary sequencer. Following criteria were employed to make the selection of the enzyme combinations: no partial digestion, no star activity, an average number of 35–70 bands per clone and a relatively even size distribution of the bands in a window ranging from 35 to 500 base pair (bp). As a result, the enzyme combination of *Bam*H I/*Hin*d III/*Hae* III was selected for generation of fingerprints from the BIBAC library in this study.

The Upland cotton BIBAC clones arrayed in 384-well microtiter plates were inoculated into 96-deep well plates, with each well containing 1.0 ml TB (Terrific Broth) medium plus 12.5 µg/ml tetracycline using a 96-pin replicator (BOEKEL, Feasterville, PA, USA). The 96-deep well plates were sealed with air-permeable seals (Excel Scientific, Wrightwood, CA, USA) and incubated in shaker at 37°C, 300 rpm for 18 h. The cultures were then centrifuged at 3,000 *g* for 10 min in a Beckman bench-top centrifuge to harvest the cells. BIBAC DNA was isolated using a modified alkaline lysis method [51], dissolved in 50 µl TE (10 mM Tris-HCl, pH 8.0, 1 mM EDTA, pH 8.0) containing 8 U/ml RNaseA and 320 U/ml RNase T1 (Applied Biosystems, Foster City, CA, USA), and stored at −20°C before use. DNA was digested and end-labeled in a reaction containing 50 mM NaCl, 10 mM Tris-HCl, 10 mM MgCl_2_, 1.0 mM dithiothreitol, pH 8.0, 1.0 mM dNTP, 1.0 µg/µl BSA, 1 U each of *Bam*H I, *Hin*d III, and *Hae* III (New England Biolabs, Ipswich, MA, USA), 0.3 U *Taq* FS and 6.0 µM HEX-ddATP or NED-ddATP. The reaction was incubated at 37°C for 90 min, followed by further incubation at 65°C for 45 min. The BIBAC DNA labeled with two fluorescent dyes, HEX-ddATP or NED-ddATP, were combined, purified and dissolved in a mixture of 9.8 µl of Hi-Di formamide with 0.2 µl of the internal GeneScan-500 Rox size standard (Applied Biosystems). DNA was denatured and analyzed on ABI 3100 Genetic Analyzer (Applied Biosystems) using the default GeneScan module. A total of 76,800 cotton BIBAC clones were fingerprinted. BIBAC fingerprint fragment sizes were determined and collected using the ABI Data Collection Program (Applied Biosystems). Data were processed and transformed into “bands” files using ABI-ExportTabularData (http://www.chromosomelab.dk/mlpa/download_ABI-ExportTabularData.html) and an automatic algorithm program SeqDisplayer (unpublished). Several quality checks were applied to the fingerprints, with vector band peaks being removed, sample-empty wells removed, background peaks identified and removed, and off-scale bands with peak heights greater than 6,000 removed. Only the bands of a clone between 35 and 500 bp were selected for physical map assembly.

### BIBAC contig physical map assembly, BES incorporation and MTP identification

The computer program FPC [52] V9.3 (http://www.agcol.arizona.edu/software/fpc/) was used to assemble the physical map contigs from the BIBAC fingerprints. Tolerance, the window size in which two restriction fragments are considered as equivalent, was set by examining the average 95% confidence interval for the realized mean size deviation of the pCLD04541 vector fragments from their true sizes as described previously [21], [43]. On the other hand, tolerances of 2–10 were tested using the entire fingerprint dataset to determine the parameter desirable for automatic contig assembly. Consequently, a tolerance of 4 was finally selected. Cutoff values (probability threshold that different fingerprint bands match by coincidence) of 1e-02 - 1e-20 were tested using the entire fingerprint dataset. The resultant numbers of contigs, singletons, and Q-clones were plotted against each other. At higher stringencies (cutoffs = 1e-09 - 1e-20), chimeric contigs were split and Q-clones were reduced, but the number of singletons increased drastically. At lower stringencies (cutoffs = 1e-02 - 1e-03), a smaller number of contigs and larger contigs were obtained, but a larger number of clones fell in the Q-clone category. The relationship among the three factors, number of contigs, number of Q-clones and number of singletons, is shown in **Figure S1**. It is apparent from **Figure S1** that a cutoff value of approximately 1e-04 to 1e-06 resulted in reasonably low numbers for all three outputs, suggesting a high quality contig assembly. On the basis of these results, a tolerance of 4 and cutoff of 1e-05 were ultimately selected for initial contig assembly.

In our previous study [35], we have generated a total of 9,711 BESs from the Upland cotton BIBAC library using the Sanger sequencing method. The BESs were incorporated into the physical map contigs manually.

Identifying MTP clones is to pick the set of minimally overlapping clones that span an entire contig and then the entire physical map. The MTP picking function of FPC V9.3 was used to identify the MTP clones in the cotton contig physical map. Two steps were involved in the process: 1) finding a set of minimally overlapping clone pairs, and 2) picking a contiguous path of overlapping clone pairs through a contig.

### Contig verification, gene-containing contig identification and subgenome-specific contig sorting

High-density clone filters were prepared from the 76,800 BIBAC clones assembled into the Upland cotton physical map as described previously [35], [43], [53]. The high-density clone filters was hybridized with 13 gene-specific overgo probes to further verify the map contigs and to identify the BIBAC contigs containing the loci of genes significant to fiber development, cellulose biosynthesis, seed fatty acid metabolism, cell wall biogenesis and cotton host-nematode interaction (**Table S1**). The library filters were also hybridized with the probes made from the PCR products of three A-and D-subgenome-specific, interspersed repetitive elements [36], [37] to test the feasibility of genome physical mapping of polyploids from BACs and/or BIBACs by fingerprint analysis (**Table S1**). All oligos either used for overgo hybridization or PCR were tested in advance using BLAT for a unique alignment with the targeted sequences in the NCBI database. Probe labeling and hybridization were performed as previously described [35], [53]–[55].

## Supporting Information

Figure S1 **Determination of optimal cutoff values.** A series of cutoff values ranging from 1e-02 to 1e-20 with a tolerance of 4 was tested for automatic contig assembly. Filled triangles indicate the number of contigs, open circles indicate the number of Q-clones and filled circles indicate the number of singletons. A cutoff value of 1e-05 was selected and used in the ultimate physical map assembly, based on the criterion that the three factors are all minimal.
(PDF)

Table S1 **Gene-specific overgos and subgenome-specific interspersed repeat elements used for the Upland cotton cv. TM-1 BIBAC library screening.**
(PDF)

Table S2 **The BIBAC physical map of the Upland cotton cv. TM-1 genome.**
(PDF)

Table S3 **Positive clones of A-subgenome specific probes, pXP128 and pXP137, and D-subgenome specific probe, pXP195.**
(PDF)
