# Supplementary material for: Genome Physical Mapping of Polyploids: A BIBAC Physical Map of Cultivated Tetraploid Cotton, Gossypium hirsutum L
Source: PLoS One. 2012 Mar 16;7(3):e33644. doi: 10.1371/journal.pone.0033644 (PMC3306275; doi:10.1371/journal.pone.0033644)
Supplement: Figure S1 — Determination of optimal cutoff values. A series of cutoff values ranging from 1e-02 to 1e-20 with a tolerance of 4 was tested for automatic contig assembly. Filled triangles indicate the number of contigs, open circles indicate the number of Q-clones and filled circles indicate the number of singletons. A cutoff value of 1e-05 was selected and used in the ultimate physical map assembly, based on the criterion that the three factors are all minimal. (PDF) [file pone.0033644.s001.pdf]

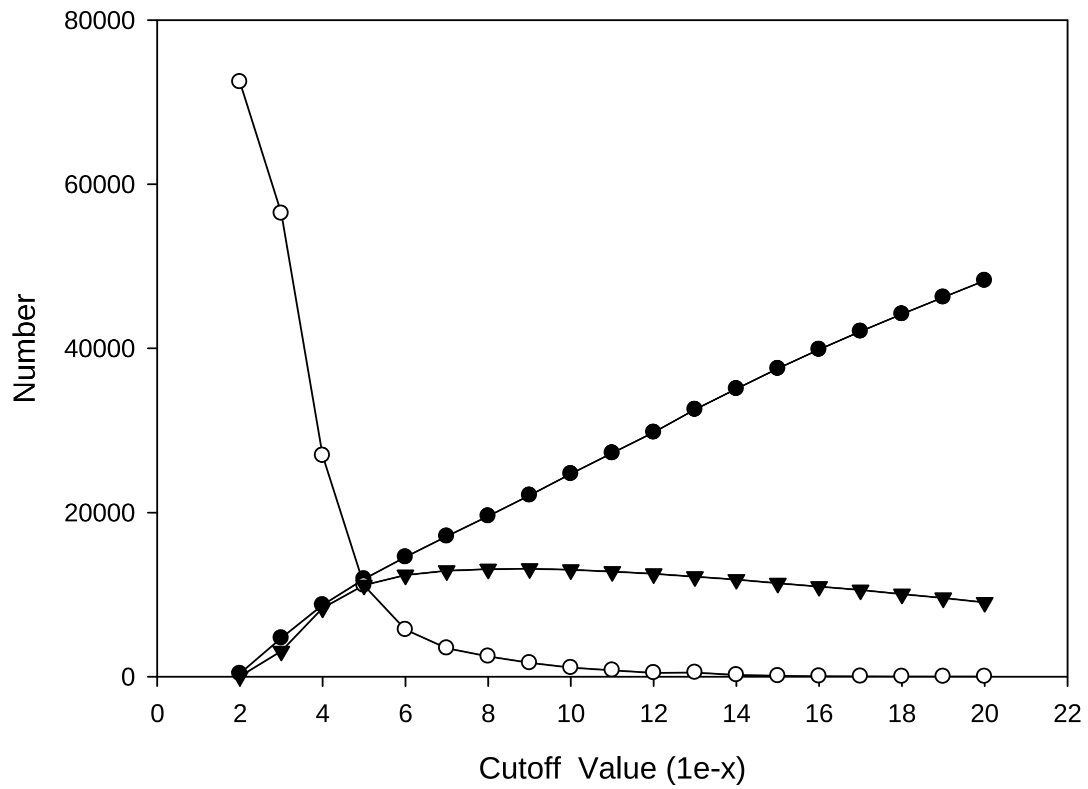

Figure S1. Determination of optimal cutoff values. A series of cutoff values ranging from 1e-02 to 1e-20 with a tolerance of 4 was tested for automatic contig assembly. Filled triangles indicate the number of contigs, open circles indicate the number of questionable clones (Q-clones) and filled circles indicate the number of singletons. A cutoff value of 1e-05 was selected and used in the ultimate physical map assembly, based on the criterion that the three factors are all minimal.
