# Supplementary material for: Genome Physical Mapping of Polyploids: A BIBAC Physical Map of Cultivated Tetraploid Cotton, Gossypium hirsutum L
Source: PLoS One. 2012 Mar 16;7(3):e33644. doi: 10.1371/journal.pone.0033644 (PMC3306275; doi:10.1371/journal.pone.0033644)
Supplement: Table S1 — Gene-specific overgos and subgenome-specific interspersed repeat elements used for the Upland cotton cv. TM-1 BIBAC library screening. (PDF) [file pone.0033644.s002.pdf]

Table S1. Gene-specific overgos and subgenome-specific interspersed repeat elements used for the Upland cotton cv. TM-1 BIBAC library screening.

Gene/ clone	GenBank Acc. No.	Annotation	Primer (Forward)	Primer (Reverse)
CelA1	HQ143024.1	Cellulose synthase A1	TGTCGTTGCCGGATTCTCCGATGC	GTACCCTTTGTTGAGGGCATCGGA
CelA3	HQ143030.1	Cellulose synthase A3	CGGGAATCAGTCTTGCCCTCAATG	CTTGTATCTGGTCTTGCATTGAGG
CelA6	GQ200733.1	Cellulose synthase catalytic subunit A3	TGTCATGGCTGGCGATCTGTGTAC	GTCTTTTAGGTATGCAGTACACAG
MYBB	AF034130.1	MYB-like DNA-binding domain protein	AGTTGGGAACTTATGGCTAAATTGTA	GCCAGTTTTTTAGTTACAATTTAGCC
MYBT2	AY366352.1	MYB-like transcription factor 2	TTGTGCATCTTCTTCAAAGCAAAC	TGTTGGAGTTGTTGCTGTTTGCTT
MIC3	GQ231916.1	*Meloidogyne*-induced cotton protein 3	TTTTGTAGCAGAAGAAATTAATTAATT	TGATATATAGTTTAATTAATTAATTTC
MIC1-15	EU025993	*Meloidogyne*-induced cotton protein 1-15	GTTTGGGAGGATATAGTGAGCATC	TCTGGTAGGGCTGAAGGATGCTCA
RDL1	AY633558.1	GaRDL1 gene, promoter region	TGTGGAGAAATCTTGTTCTACTTTCC	GTGTTGCATGGAATGGAAAGTAGAAC
FADO6	Y10112.2	Fatty acid desaturase omega-6	AGGCAGAATGTCGGTTCCAACGAG	TTCGGGTTTTTTTGGACTCGTTGG
GhCesA2	U58284.1	Secondary wall cellulose synthase A2	GTCTCTGAGAAACGACCAAAGATG	GCCAGCAATCACATGTCATCTTTG
GhRX3	DT048689	Irregular xylem3/cellulose synthase 7	GTTCGCGGTTATCCAAGGCTTGTT	ACCAGCTAGAACTTTCAACAAGCC
GhCesA3	AF150630.2	Primary wall cellulose synthase catalytic subunit A3	GAGTTACCGGGCCGGATGTAGAAC	CAGTTTATTCCACACTGTTCTACA
GhCes	AF150630	Unknown cellulose synthase	GAGTTACCGGGCCGGATGTAGAAC	CAGTTTATTCCACACTGTTCTACA
pXP128	AF060622.1	A-subgenome-specific, interspersed repeated element	CTAACTCATCTCCCAC	TTAAGTTTGTGTTGTTGAGC
pXP137 (pXP095)	AF060607	A-subgenome-specific, interspersed repeated element	CTCTGTACATGGAGATAT	ATTGTTGTTGTGGGAATTGT
pXP195	AF060634.1	D-subgenome-specific, interspersed repeated element	TCTCGAGGAGCTGGAGACAA	TCCTTGCTAACAAGCTCTTG
