# Supplementary material for: Genome Physical Mapping of Polyploids: A BIBAC Physical Map of Cultivated Tetraploid Cotton, Gossypium hirsutum L
Source: PLoS One. 2012 Mar 16;7(3):e33644. doi: 10.1371/journal.pone.0033644 (PMC3306275; doi:10.1371/journal.pone.0033644)
Supplement: Table S2 — The BIBAC physical map of the Upland cotton cv. TM-1 genome. (PDF) [file pone.0033644.s003.pdf]

Table S2. The BIBAC physical map of the Upland cotton cv. TM-1 genome.

Contig No.	Clones in contig	Physical length (kb)	Consensus bands (CBs)	Origin of subgenome	Genes contained in the contig
1	140	3,334	946	A	
2	20	489	139	A	
3	11	687	195		
4	14	419	119		
5	112	3,433	974	A	
6	17	549	156	A	
7	8	408	116	A	
8	77	2,030	576		
9	10	260	74		
10	15	461	131		
11	18	412	117		
12	34	941	267	A	
13	76	1,656	470	A	
14	59	1,575	447	A	
15	58	1,406	399	A	
16	12	246	70		
17	83	2,358	669	A	
18	7	172	49		
19	10	306	87		
20	12	394	112		
21	64	1,935	549	A	
22	14	296	84		
23	93	3,102	880	A	
24	16	472	134		
25	34	1,343	381		
26	15	648	184		
27	163	4,402	1,249	A	
28	147	4,931	1,399	A	
29	18	500	142		
30	3	119	34		
31	15	232	66		
32	13	377	107		
33	37	1,050	298	A	
34	15	902	256		
35	18	334	95	D	
36	16	356	101		
37	41	1,138	323		
38	27	719	204	A	
39	14	348	99		
40	6	236	67	A	
41	46	1,226	348	A	
42	7	218	62		
43	7	348	99		
44	77	2,393	679		
45	6	229	65		
46	14	440	125		
47	104	3,299	936	A	
48	8	299	85	A	

49	59	1,487	422	A
50	30	796	226	A
51	31	1,015	288	D
52	69	2,016	572	A
53	14	401	114	A
54	6	253	72	A
55	27	870	247	
56	30	1,001	284	
57	90	3,221	914	A
58	50	1,360	386	A
59	7	289	82	D
60	103	2,241	636	A
61	67	1,635	464	
62	13	482	137	
63	43	1,684	478	A
64	111	3,024	858	A
65	17	458	130	
66	10	271	77	
67	41	1,378	391	A
68	52	1,216	345	A
69	41	1,508	428	
70	56	1,780	505	A
71	84	3,274	929	
72	36	1,265	359	
73	16	694	197	A
74	13	433	123	
75	28	747	212	A
76	125	3,556	1,009	A
77	38	697	198	A
78	79	2,097	595	A
79	31	1,128	320	
80	37	1,128	320	A
81	42	1,656	470	A
82	5	176	50	
83	69	1,716	487	A
84	44	1,029	292	
85	20	440	125	
86	13	310	88	D
87	11	380	108	
88	25	987	280	
89	7	289	82	A
90	29	1,272	361	A
91	21	1,216	345	
92	94	2,749	780	A
93	34	1,247	354	
94	11	243	69	
95	15	391	111	A
96	32	1,032	293	A
97	46	1,392	395	
98	19	641	182	
99	15	504	143	
100	27	905	257	

101	31	1,025	291	
102	15	430	122	D
103	10	260	74	
104	23	757	215	
105	9	514	146	
106	30	768	218	A
107	29	944	268	
108	33	768	218	D
109	37	979	278	
110	37	1,223	347	A
111	28	1,015	288	
112	59	2,136	606	
113	31	831	236	A
114	58	2,397	680	A
115	78	2,315	657	A
116	15	556	158	
117	76	2,530	718	A
118	42	951	270	D
119	40	1,371	389	A
120	37	1,187	337	
121	63	2,284	648	A
122	23	652	185	
123	20	556	158	A
124	53	2,315	657	
125	8	264	75	
126	8	243	69	
127	6	274	78	
128	28	1,128	320	
129	79	2,076	589	A
130	98	2,111	599	D
131	64	2,256	640	A
132	10	292	83	
133	3	183	52	A
134	6	232	66	
135	15	479	136	
136	34	1,166	331	A
137	53	1,706	484	
138	105	3,352	951	A
139	17	493	140	A
140	57	2,291	650	A
141	24	779	221	
142	44	1,357	385	A
143	13	257	73	
144	29	779	221	
145	33	909	258	
146	77	2,488	706	A
147	13	475	135	A
148	41	1,625	461	A
149	22	645	183	A
150	14	796	226	
151	50	1,142	324	A
152	65	1,677	476	A

153	14	387	110	
154	36	1,254	356	A
155	50	2,026	575	A
156	10	310	88	
157	161	4,660	1,322	A
158	6	193	55	
159	18	408	116	A
160	43	948	269	A
161	8	271	77	
162	60	1,981	562	
163	17	708	201	A
164	8	310	88	A
165	27	1,240	352	A
166	24	733	208	A
167	4	215	61	
168	21	468	133	
169	86	2,273	645	A
170	39	683	194	D
171	7	331	94	
172	24	775	220	
173	36	673	191	A
174	34	1,279	363	
175	42	1,399	397	A
176	30	923	262	A
177	13	673	191	
178	20	659	187	
179	52	2,100	596	
180	18	630	179	
181	35	1,212	344	A
182	28	715	203	
183	11	461	131	
184	6	232	66	A
185	31	1,335	379	
186	54	2,079	590	A
187	149	4,504	1,278	A
188	52	1,790	508	
189	9	366	104	
190	46	1,462	415	A
191	68	2,217	629	D
192	11	380	108	
193	42	1,392	395	A
194	40	1,279	363	
195	23	810	230	
196	52	1,825	518	D
197	187	6,380	1,810	A
198	17	627	178	
199	27	1,170	332	
200	62	1,942	551	
201	34	884	251	
202	11	504	143	
203	47	1,247	354	
204	11	324	92	

205	41	1,491	423	A
206	7	313	89	
207	20	1,085	308	
208	69	2,291	650	A
209	4	260	74	
210	12	317	90	
211	30	1,124	319	
212	22	786	223	A
213	9	250	71	
214	18	556	158	
215	23	694	197	A
216	50	1,543	438	A
217	21	468	133	
218	57	2,308	655	A
219	9	472	134	
220	10	482	137	
221	12	373	106	A
222	27	849	241	A
223	65	2,361	670	A
224	4	193	55	
225	6	331	94	A
226	16	532	151	
227	13	412	117	
228	114	3,581	1,016	A
229	44	1,311	372	A
230	8	204	58	
231	16	641	182	
232	22	539	153	
233	38	1,494	424	A
234	60	2,146	609	A
235	75	2,058	584	A
236	19	789	224	A
237	11	370	105	
238	29	1,057	300	
239	6	310	88	
240	5	320	91	
241	10	313	89	
242	6	190	54	
243	33	1,142	324	
244	22	588	167	
245	32	1,276	362	A
246	19	824	234	A
247	6	271	77	
248	9	320	91	
249	32	1,011	287	A
250	30	712	202	
251	12	239	68	
252	14	317	90	
253	103	3,606	1,023	A
254	76	2,062	585	A
255	30	920	261	
256	18	556	158	

257	10	454	129		
258	12	331	94		*GhCesA3, Ces*
259	11	303	86		
260	7	289	82		
261	54	1,540	437	A	
262	23	507	144	D	
263	46	1,339	380	A	
264	20	715	203	A	
265	110	4,124	1,170	A	
266	100	3,539	1,004	A	
267	43	1,861	528	A	
268	16	412	117		
269	6	236	67		
270	30	1,011	287	D	
271	19	708	201		
272	35	1,124	319		
273	122	3,419	970	D	
274	23	803	228	A	
275	19	955	271		
276	31	1,156	328	A	
277	8	370	105		
278	5	264	75		
279	6	257	73		
280	34	1,166	331	A	
281	33	1,452	412	A	
282	19	835	237	A	
283	3	165	47	D	
284	6	243	69		
285	149	5,192	1,473	A	
286	12	267	76		
287	37	1,378	391	A	
288	4	260	74		
289	12	482	137		
290	31	1,061	301	A	
291	6	165	47		
292	17	733	208		
293	93	2,798	794	A	
294	17	423	120	D	
295	20	634	180		
296	20	835	237		
297	7	155	44		
298	129	4,582	1,300	A	
299	8	359	102		
300	7	162	46		
301	18	659	187	A	
302	7	437	124		
303	24	1,068	303	A	
304	29	955	271	A	
305	11	341	97		
306	17	585	166		
307	50	1,343	381		
308	7	334	95		

309	33	1,427	405	A
310	23	807	229	
311	63	2,115	600	A
312	32	1,078	306	A
313	10	458	130	
314	111	2,961	840	A
315	12	437	124	
316	3	91	26	A
317	56	1,847	524	A
318	16	514	146	
319	17	740	210	
320	49	1,554	441	
321	33	962	273	
322	8	264	75	A
323	15	786	223	A
324	65	2,319	658	A
325	4	179	51	
326	31	1,251	355	A
327	7	246	70	
328	7	190	54	
329	46	1,543	438	A
330	44	1,519	431	
331	10	468	133	
332	7	264	75	
333	13	447	127	
334	6	193	55	
335	51	1,695	481	A
336	57	1,896	538	
337	6	363	103	
338	109	3,503	994	A
339	50	1,519	431	A
340	7	380	108	
341	38	1,261	358	A
342	41	1,484	421	
343	21	1,170	332	
344	64	1,963	557	A
345	42	1,113	316	A
346	23	951	270	A
347	61	1,974	560	A
348	52	1,582	449	A
349	36	1,061	301	A
350	41	1,491	423	A
351	33	1,018	289	
352	37	1,307	371	A
353	45	1,314	373	
354	44	1,244	353	A
355	30	990	281	
356	25	937	266	A
357	81	2,671	758	A
358	44	1,459	414	
359	31	1,586	450	
360	20	789	224	

361	57	1,695	481	A	
362	35	835	237		
363	13	292	83		
364	35	1,367	388	A	*CelA6*
365	14	560	159		
366	104	1,670	474	A	
367	83	2,287	649	A	
368	16	652	185		
369	5	236	67		
370	6	257	73		
371	7	207	59		
372	40	1,170	332		
373	31	1,128	320		
374	19	793	225		
375	17	571	162		
376	34	1,068	303		
377	64	1,878	533	A	
378	46	1,480	420	A	
379	103	2,823	801	A	
380	21	722	205	A	
381	16	800	227		
382	31	1,092	310	A	
383	24	881	250		
384	57	1,868	530	A	
385	22	514	146	A	
386	81	2,911	826		
387	5	334	95		
388	19	627	178		
389	6	239	68		
390	10	528	150		
391	34	821	233	D	
392	17	482	137	A	
393	13	423	120		
394	30	1,071	304	A	
395	29	948	269	A	
396	10	408	116		
397	33	1,230	349	A	
398	3	215	61		
399	21	1,064	302		
400	19	616	175		
401	9	260	74		
402	5	207	59	D	
403	17	518	147		
404	46	1,942	551		
405	6	246	70		
406	7	444	126	A	
407	22	846	240	A	
408	40	1,667	473	A	
409	22	965	274		
410	33	972	276	A	
411	105	3,458	981	A	
412	18	680	193		

413	32	1,187	337	
414	46	1,381	392	
415	44	1,413	401	A
416	6	218	62	
417	28	955	271	A
418	19	697	198	
419	44	1,515	430	A
420	27	1,110	315	A
421	19	645	183	A
422	47	1,632	463	A
423	91	2,657	754	
424	18	567	161	
425	47	1,417	402	A
426	41	1,304	370	A
427	72	2,495	708	A
428	32	923	262	A
429	14	634	180	
430	84	2,830	803	A
431	5	257	73	
432	19	905	257	
433	14	366	104	
434	26	920	261	
435	28	782	222	
436	45	1,462	415	A
437	91	2,664	756	A
438	10	560	159	A
439	21	581	165	
440	10	232	66	
441	54	2,093	594	A
442	35	1,265	359	
443	3	211	60	
444	11	387	110	
445	44	1,106	314	
446	48	1,727	490	
447	72	2,446	694	
448	27	863	245	
449	36	1,350	383	A
450	23	824	234	A
451	37	1,357	385	A
452	8	232	66	
453	39	1,128	320	A
454	114	3,320	942	A
455	3	215	61	A
456	8	472	134	
457	31	1,254	356	
458	9	363	103	
459	46	1,596	453	A
460	33	1,240	352	
461	6	285	81	
462	70	814	231	A
463	10	652	185	A
464	45	1,603	455	A

465	33	1,378	391	
466	34	1,223	347	
467	15	458	130	
468	4	211	60	
469	11	475	135	A
470	60	1,878	533	A
471	72	2,298	652	A
472	18	669	190	
473	8	317	90	
474	58	2,051	582	
475	21	712	202	A
476	64	2,939	834	A
477	5	193	55	
478	21	754	214	A
479	4	229	65	
480	23	585	166	
481	13	327	93	D
482	27	1,018	289	A
483	48	1,498	425	
484	15	609	173	
485	26	821	233	
486	8	334	95	
487	4	186	53	
488	4	243	69	
489	18	701	199	
490	18	623	177	
491	13	680	193	
492	34	789	224	A
493	8	373	106	A
494	63	1,836	521	A
495	87	3,214	912	A
496	21	690	196	A
497	5	253	72	
498	10	458	130	
499	18	592	168	
500	47	1,424	404	A
501	21	807	229	
502	109	2,717	771	A
503	12	465	132	A
504	8	324	92	A
505	39	1,212	344	
506	3	204	58	
507	12	616	175	A
508	17	564	160	A
509	48	1,621	460	A
510	7	627	178	
511	15	687	195	
512	45	1,720	488	A
513	9	250	71	
514	36	1,187	337	A
515	28	1,011	287	A
516	24	1,050	298	

517	62	2,167	615	A
518	14	634	180	A
519	67	2,100	596	
520	42	1,128	320	
521	93	2,999	851	A
522	10	440	125	
523	15	786	223	D
524	4	186	53	
525	13	426	121	
526	22	1,145	325	A
527	33	1,230	349	A
528	7	225	64	
529	16	627	178	
530	15	317	90	D
531	10	475	135	A
532	57	1,727	490	A
533	18	920	261	
534	65	2,079	590	A
535	19	881	250	
536	13	475	135	
537	27	1,078	306	
538	6	303	86	
539	23	1,089	309	A
540	24	743	211	
541	49	1,720	488	A
542	12	542	154	
543	6	172	49	
544	8	373	106	
545	29	1,053	299	
546	71	2,164	614	A
547	101	3,331	945	A
548	7	341	97	A
549	18	740	210	
550	25	814	231	A
551	29	807	229	A
552	106	3,581	1,016	A
553	3	172	49	
554	43	1,159	329	
555	34	1,177	334	
556	67	2,238	635	D
557	54	1,825	518	A
558	10	465	132	A
559	7	433	123	
560	13	528	150	
561	45	1,032	293	
562	28	1,240	352	
563	87	2,545	722	A
564	16	800	227	
565	20	567	161	
566	16	306	87	D
567	16	627	178	
568	7	377	107	

569	30	1,163	330	A
570	25	1,015	288	A
571	43	1,702	483	A
572	68	2,146	609	A
573	20	779	221	A
574	4	186	53	
575	25	807	229	
576	17	743	211	A
577	35	1,008	286	
578	11	528	150	
579	82	2,763	784	A
580	8	415	118	
581	96	2,915	827	
582	36	1,505	427	
583	110	3,919	1,112	A
584	16	535	152	
585	6	148	42	
586	43	1,575	447	A
587	7	197	56	
588	47	1,829	519	
589	10	331	94	A
590	9	387	110	
591	13	645	183	A
592	17	676	192	A
593	8	218	62	
594	7	317	90	
595	10	359	102	
596	6	278	79	
597	6	236	67	A
598	13	655	186	A
599	13	518	147	
600	60	2,125	603	A
601	5	267	76	
602	88	2,710	769	A
603	84	2,626	745	
604	21	705	200	
605	60	2,033	577	A
606	34	1,297	368	A
607	4	186	53	
608	9	405	115	
609	14	500	142	A
610	17	662	188	
611	29	1,103	313	D
612	33	934	265	A
613	29	972	276	A
614	6	250	71	
615	3	197	56	
616	13	260	74	D
617	11	546	155	
618	11	384	109	
619	50	1,223	347	A
620	30	1,075	305	A

621	27	1,205	342	
622	9	387	110	A
623	14	405	115	
624	10	310	88	
625	41	1,251	355	A
626	6	232	66	
627	64	1,854	526	
628	17	528	150	A
629	74	2,213	628	A+D
630	9	394	112	
631	37	1,392	395	A
632	17	1,050	298	
633	15	645	183	
634	17	680	193	A
635	33	1,187	337	D
636	52	1,674	475	A
637	7	405	115	A
638	14	602	171	
639	37	1,082	307	
640	10	486	138	
641	9	387	110	
642	37	1,388	394	
643	37	831	236	A
644	27	1,166	331	A
645	15	662	188	
646	8	250	71	
647	23	997	283	
648	19	655	186	
649	16	638	181	
650	28	1,117	317	A
651	50	2,062	585	
652	7	158	45	
653	19	902	256	A
654	3	229	65	
655	4	141	40	
656	10	454	129	
657	35	1,187	337	A
658	8	380	108	
659	10	415	118	
660	5	292	83	
661	46	1,343	381	
662	14	500	142	A+D
663	56	1,914	543	A
664	14	528	150	
665	114	3,387	961	A
666	24	891	253	A
667	26	641	182	A
668	62	2,192	622	A
669	3	218	62	
670	72	2,562	727	A
671	46	1,660	471	A
672	13	546	155	

673	22	779	221	A
674	36	1,663	472	A
675	35	1,261	358	A
676	14	359	102	A
677	20	719	204	
678	47	2,076	589	A
679	18	814	231	
680	37	1,286	365	
681	22	1,128	320	A
682	22	719	204	
683	7	405	115	A
684	45	1,512	429	
685	16	659	187	
686	5	211	60	
687	22	641	182	
688	7	236	67	
689	3	215	61	
690	30	1,061	301	
691	32	902	256	
692	11	578	164	
693	20	934	265	
694	8	148	42	
695	56	1,254	356	A
696	5	158	45	
697	21	764	217	
698	14	504	143	
699	5	356	101	
700	8	423	120	A
701	46	2,097	595	A
702	14	546	155	
703	15	564	160	
704	39	1,240	352	A
705	24	884	251	
706	72	2,382	676	A
707	61	1,808	513	A
708	26	877	249	
709	105	2,834	804	A
710	18	715	203	
711	33	1,092	310	A
712	15	638	181	
713	20	669	190	A
714	5	451	128	
715	7	271	77	
716	11	401	114	
717	27	1,131	321	
718	4	158	45	
719	9	486	138	
720	12	461	131	
721	19	602	171	
722	55	2,107	598	A
723	40	1,628	462	A
724	5	320	91	

725	4	317	90	
726	50	1,501	426	
727	17	458	130	
728	12	578	164	A
729	16	793	225	
730	19	736	209	A
731	51	1,543	438	D
732	20	701	199	A
733	44	1,536	436	A
734	4	193	55	
735	7	222	63	
736	16	433	123	
737	4	200	57	A
738	27	807	229	A
739	12	461	131	
740	16	398	113	
741	70	2,136	606	A
742	43	1,452	412	A
743	10	423	120	
744	14	687	195	
745	15	514	146	
746	22	976	277	A
747	7	306	87	
748	70	1,099	312	A
749	10	641	182	
750	28	1,036	294	A
751	31	972	276	A
752	40	1,117	317	
753	9	535	152	
754	7	292	83	
755	10	405	115	
756	39	1,575	447	
757	14	497	141	
758	38	1,399	397	A
759	13	511	145	
760	32	1,209	343	A
761	12	440	125	
762	14	623	177	A
763	78	2,548	723	A
764	10	493	140	
765	51	2,051	582	D
766	4	229	65	
767	20	962	273	
768	19	990	281	
769	12	585	166	D
770	25	708	201	
771	126	3,599	1,021	A
772	8	356	101	
773	22	990	281	A
774	92	1,998	567	A
775	9	303	86	
776	17	1,015	288	A

777	10	514	146	
778	6	243	69	A
779	14	482	137	
780	6	461	131	
781	45	1,410	400	
782	113	2,749	780	A
783	13	486	138	
784	15	652	185	
785	26	1,113	316	A
786	21	888	252	
787	9	370	105	
788	29	1,106	314	
789	8	620	176	
790	35	1,480	420	A
791	8	363	103	
792	12	317	90	A
793	33	1,173	333	A
794	11	423	120	
795	23	814	231	
796	47	1,804	512	A
797	20	1,166	331	
798	51	1,575	447	A
799	6	190	54	
800	63	2,280	647	A
801	40	1,346	382	A
802	15	556	158	
803	22	528	150	
804	46	1,565	444	A
805	6	179	51	
806	6	655	186	
807	40	1,586	450	A
808	95	3,176	901	A
809	29	1,018	289	A
810	85	2,693	764	A
811	44	1,314	373	A
812	8	363	103	
813	13	764	217	A
814	4	190	54	
815	39	1,198	340	
816	31	987	280	
817	30	1,025	291	
818	37	1,385	393	A
819	5	267	76	
820	63	1,667	473	A
821	16	648	184	
822	6	377	107	A
823	7	451	128	
824	3	158	45	
825	19	560	159	
826	46	1,677	476	
827	3	236	67	
828	9	341	97	

829	15	726	206	A
830	14	842	239	
831	34	994	282	A
832	6	225	64	
833	13	613	174	A
834	42	1,716	487	A
835	5	204	58	
836	16	401	114	D
837	3	232	66	
838	24	800	227	
839	19	979	278	
840	12	542	154	
841	13	401	114	A
842	36	1,286	365	A
843	24	659	187	
844	10	327	93	
845	5	179	51	A
846	8	514	146	
847	39	1,402	398	A
848	7	366	104	
849	5	229	65	
850	8	500	142	
851	79	2,372	673	A
852	4	155	44	
853	9	405	115	
854	33	1,061	301	
855	57	2,026	575	
856	14	345	98	
857	5	260	74	
858	8	348	99	
859	25	712	202	
860	6	257	73	
861	23	909	258	A
862	6	197	56	
863	24	726	206	A
864	3	130	37	
865	7	278	79	A
866	17	687	195	
867	18	514	146	
868	18	669	190	A
869	32	1,068	303	
870	82	2,781	789	A
871	16	712	202	A
872	16	701	199	A
873	5	193	55	
874	40	1,163	330	A
875	4	200	57	
876	9	285	81	
877	5	299	85	A
878	7	479	136	
879	32	1,283	364	
880	21	856	243	

881	7	359	102	
882	50	1,854	526	A
883	7	299	85	
884	69	2,125	603	A
885	5	176	50	
886	22	853	242	
887	5	253	72	
888	7	264	75	
889	63	2,097	595	A
890	4	137	39	A
891	48	1,790	508	
892	8	475	135	
893	14	567	161	
894	31	1,293	367	
895	21	764	217	
896	22	757	215	
897	38	1,145	325	
898	19	549	156	
899	6	257	73	
900	48	1,445	410	A
901	11	359	102	
902	17	634	180	
903	24	623	177	A
904	5	211	60	
905	29	1,092	310	
906	20	927	263	A
907	11	444	126	
908	8	282	80	
909	18	567	161	
910	10	366	104	
911	4	225	64	
912	62	2,213	628	A
913	17	511	145	
914	6	528	150	
915	38	1,695	481	A
916	29	1,233	350	
917	42	1,434	407	
918	16	715	203	A
919	32	1,022	290	
920	14	493	140	
921	21	641	182	A
922	4	222	63	
923	5	264	75	
924	32	1,311	372	
925	16	556	158	
926	34	1,388	394	A
927	42	1,202	341	A
928	14	740	210	
929	23	958	272	A
930	30	1,198	340	
931	22	673	191	A
932	25	690	196	D

933	8	289	82		
934	4	193	55		
935	10	338	96	A	
936	10	553	157		
937	19	771	219		
938	9	415	118		
939	22	775	220		
940	35	1,600	454		
941	16	683	194		
942	9	296	84		
943	35	1,466	416	A	
944	31	1,078	306		
945	7	310	88		
946	13	440	125		
947	19	701	199	A	
948	5	260	74		
949	11	652	185		
950	39	1,684	478		
951	3	204	58		
952	10	394	112		
953	16	567	161	A	
954	43	1,318	374		
955	5	274	78		
956	6	331	94	A	
957	8	486	138		
958	8	373	106		
959	6	327	93		
960	39	1,350	383		
961	37	1,484	421		
962	11	497	141	A	
963	6	426	121	A	
964	16	874	248	A	
965	7	246	70		
966	11	669	190	A	
967	23	764	217		
968	24	1,103	313	A	
969	21	916	260		
970	10	348	99	A	
971	17	525	149		
972	5	190	54		
973	8	327	93		
974	13	595	169	A	
975	94	1,899	539	A	
976	22	828	235		
977	14	838	238		
978	25	729	207	A	
979	23	884	251		*CelA3*
980	15	1,036	294		
981	23	888	252		
982	78	1,815	515	A	
983	39	1,283	364		
984	21	712	202		

985	16	733	208	
986	3	151	43	
987	6	260	74	
988	26	863	245	A
989	28	712	202	
990	7	627	178	
991	31	1,177	334	A
992	24	712	202	A
993	10	733	208	
994	18	740	210	
995	3	225	64	
996	19	669	190	
997	33	1,205	342	
998	34	1,216	345	D
999	21	1,043	296	
1000	5	243	69	
1001	22	828	235	A
1002	10	253	72	
1003	24	771	219	A+D
1004	4	313	89	
1005	4	179	51	
1006	22	898	255	A
1007	10	479	136	
1008	34	1,304	370	A
1009	14	535	152	
1010	16	535	152	
1011	22	810	230	
1012	28	1,046	297	D
1013	5	232	66	
1014	8	331	94	
1015	21	888	252	A
1016	7	186	53	
1017	4	190	54	
1018	20	662	188	
1019	4	218	62	
1020	6	246	70	
1021	3	215	61	
1022	17	814	231	
1023	25	1,053	299	
1024	30	1,117	317	
1025	8	398	113	
1026	30	810	230	A
1027	75	1,945	552	A
1028	92	3,673	1,042	A
1029	10	504	143	
1030	25	951	270	D
1031	9	426	121	
1032	43	1,512	429	A
1033	6	243	69	A
1034	7	253	72	
1035	20	860	244	A
1036	3	243	69	

1037	30	1,184	336	A
1038	16	627	178	A
1039	17	627	178	
1040	8	359	102	
1041	7	292	83	
1042	10	479	136	
1043	6	264	75	
1044	22	895	254	
1045	14	1,001	284	A
1046	3	197	56	
1047	30	1,131	321	A
1048	5	197	56	
1049	12	430	122	
1050	21	701	199	
1051	36	1,131	321	
1052	19	475	135	A
1053	15	532	151	
1054	16	482	137	
1055	4	222	63	
1056	36	1,304	370	
1057	4	282	80	
1058	67	2,865	813	A
1059	25	860	244	A
1060	5	239	68	
1061	10	204	58	
1062	25	1,343	381	A
1063	20	1,022	290	
1064	67	2,266	643	A
1065	24	1,138	323	
1066	13	493	140	A
1067	32	1,269	360	A
1068	33	655	186	A
1069	27	1,120	318	A
1070	5	215	61	
1071	21	669	190	A
1072	6	306	87	
1073	8	423	120	
1074	37	1,773	503	A
1075	7	348	99	
1076	5	292	83	
1077	6	193	55	
1078	15	733	208	A
1079	63	2,012	571	
1080	6	257	73	
1081	52	1,744	495	A
1082	40	1,445	410	
1083	3	200	57	
1084	16	842	239	A
1085	77	2,326	660	A
1086	44	1,758	499	
1087	5	165	47	
1088	4	218	62	

1089	18	761	216	A	
1090	70	2,671	758	D	*GhIRX3*
1091	6	313	89		
1092	3	215	61		
1093	3	225	64		
1094	25	824	234		
1095	42	1,501	426	A	
1096	21	838	238	A	
1097	18	1,198	340		
1098	28	1,223	347	A	
1099	10	398	113		
1100	82	2,661	755	A	
1101	39	1,304	370		
1102	37	1,388	394		
1103	59	1,998	567	A	
1104	29	1,240	352		
1105	9	666	189		
1106	6	260	74		
1107	32	1,187	337		
1108	14	556	158		*CelA1*
1109	18	359	102		
1110	24	1,128	320		
1111	6	271	77		
1112	64	1,949	553	A	
1113	40	1,265	359	A	
1114	14	504	143	A	
1115	7	190	54		
1116	68	2,139	607	A	
1117	14	638	181		
1118	6	264	75		
1119	28	979	278	A	
1120	14	391	111		
1121	6	356	101	A	
1122	26	870	247	A	
1123	17	1,029	292	A	
1124	54	1,226	348	D	
1125	8	394	112	A	
1126	7	363	103		
1127	17	712	202		
1128	7	207	59		
1129	5	359	102		
1130	31	556	158		
1131	11	486	138	A	
1132	60	2,520	715	A	
1133	48	1,596	453	A	
1134	10	334	95		
1135	3	215	61		
1136	9	370	105	A	
1137	14	768	218		*MYBB*
1138	39	1,180	335	A	
1139	56	1,360	386	A	
1140	9	415	118		

1141	3	246	70		
1142	8	398	113		
1143	4	204	58		
1144	4	278	79		
1145	4	243	69		
1146	9	444	126		
1147	75	2,474	702	A	
1148	5	253	72		
1149	11	803	228	A	
1150	8	352	100		
1151	20	863	245		
1152	18	888	252		
1153	6	437	124		
1154	6	292	83		
1155	32	881	250	A	
1156	58	2,079	590	A	*FADO6*
1157	39	1,469	417		
1158	40	1,272	361	A	
1159	39	1,149	326	A	
1160	17	627	178	A	
1161	6	606	172		
1162	14	500	142	A	
1163	28	1,082	307	A	
1164	3	179	51	A	
1165	6	327	93		
1166	18	578	164	A	
1167	4	137	39		
1168	38	1,684	478	A	
1169	7	271	77		
1170	12	475	135	A	
1171	71	2,354	668	A	
1172	14	719	204	A	
1173	5	271	77		
1174	30	1,166	331		
1175	3	225	64		
1176	5	250	71		
1177	29	1,142	324		
1178	7	250	71		
1179	9	338	96		
1180	39	1,371	389	D	
1181	13	419	119	A	
1182	13	486	138		
1183	85	3,165	898	A	
1184	21	606	172		
1185	13	687	195		
1186	22	701	199		
1187	3	151	43		
1188	28	1,149	326	A	
1189	17	793	225		
1190	40	891	253	D	
1191	4	186	53		
1192	33	1,149	326	A	

1193	3	257	73	
1194	3	197	56	A
1195	6	380	108	A
1196	7	299	85	
1197	11	743	211	A
1198	36	1,233	350	A
1199	37	1,667	473	
1200	47	2,048	581	
1201	3	193	55	
1202	13	736	209	
1203	20	764	217	A
1204	28	779	221	A
1205	9	306	87	
1206	3	158	45	
1207	30	965	274	D
1208	8	377	107	
1209	35	1,328	377	A
1210	18	768	218	
1211	25	1,360	386	
1212	4	186	53	
1213	26	588	167	A
1214	42	1,113	316	
1215	6	260	74	
1216	15	472	134	
1217	52	1,462	415	A
1218	18	856	243	
1219	18	648	184	
1220	4	250	71	A
1221	17	705	200	
1222	64	2,312	656	A
1223	25	958	272	A
1224	5	204	58	
1225	12	768	218	
1226	14	497	141	D
1227	13	609	173	A
1228	3	232	66	
1229	11	363	103	A
1230	43	1,410	400	A
1231	27	1,286	365	A
1232	38	1,632	463	D
1233	18	838	238	
1234	23	768	218	
1235	22	754	214	A
1236	3	169	48	
1237	21	898	255	
1238	13	412	117	A
1239	10	408	116	
1240	16	599	170	A
1241	8	285	81	
1242	4	193	55	
1243	61	1,632	463	D
1244	4	211	60	

1245	3	162	46	
1246	9	673	191	A
1247	18	775	220	A
1248	41	1,364	387	
1249	12	363	103	
1250	60	1,811	514	A
1251	5	218	62	
1252	19	867	246	
1253	11	419	119	
1254	47	1,318	374	A
1255	21	1,261	358	
1256	21	775	220	A
1257	4	285	81	
1258	20	775	220	
1259	5	239	68	
1260	28	888	252	A
1261	26	1,071	304	
1262	7	426	121	
1263	54	1,878	533	A
1264	12	465	132	
1265	8	521	148	
1266	39	1,670	474	A
1267	41	1,269	360	A
1268	3	155	44	
1269	9	310	88	
1270	17	655	186	
1271	20	683	194	
1272	12	451	128	D
1273	13	489	139	
1274	5	183	52	
1275	45	1,508	428	A
1276	19	807	229	
1277	9	398	113	A
1278	60	2,189	621	A
1279	37	1,688	479	A
1280	44	1,156	328	A
1281	6	211	60	
1282	27	853	242	
1283	13	482	137	
1284	18	676	192	
1285	5	285	81	
1286	3	271	77	A
1287	38	1,406	399	D
1288	18	652	185	
1289	13	426	121	
1290	6	250	71	
1291	17	972	276	
1292	17	990	281	A
1293	25	786	223	A
1294	39	1,561	443	D
1295	48	1,966	558	D
1296	3	246	70	

1297	16	789	224	
1298	6	373	106	A
1299	7	232	66	
1300	26	1,247	354	A
1301	7	306	87	
1302	19	592	168	
1303	31	1,216	345	A
1304	11	423	120	
1305	28	930	264	A
1306	21	934	265	A
1307	6	327	93	
1308	6	327	93	
1309	30	1,177	334	
1310	55	1,977	561	A
1311	9	440	125	
1312	16	958	272	
1313	9	356	101	
1314	24	853	242	
1315	46	1,392	395	A
1316	6	292	83	
1317	13	662	188	
1318	6	338	96	A
1319	7	274	78	
1320	14	458	130	
1321	5	296	84	
1322	5	285	81	
1323	6	250	71	
1324	16	652	185	A
1325	25	673	191	D
1326	9	539	153	
1327	28	1,046	297	A
1328	12	599	170	
1329	45	1,748	496	A
1330	4	299	85	
1331	8	391	111	
1332	22	831	236	
1333	47	1,593	452	A
1334	14	609	173	D
1335	4	267	76	
1336	13	556	158	
1337	29	1,462	415	A
1338	5	338	96	
1339	12	430	122	
1340	4	126	36	
1341	5	250	71	
1342	37	1,519	431	A
1343	5	172	49	
1344	26	786	223	
1345	7	697	198	
1346	8	296	84	A
1347	5	186	53	
1348	15	560	159	A

1349	3	190	54	D	
1350	67	2,005	569	A	
1351	7	239	68		
1352	5	278	79		
1353	39	877	249	A	
1354	9	289	82		
1355	3	236	67		
1356	13	592	168		
1357	6	222	63		
1358	4	211	60	A	
1359	86	3,197	907	A	
1360	6	225	64		
1361	5	419	119		
1362	15	655	186		
1363	15	486	138	D	
1364	4	207	59		
1365	3	289	82		
1366	38	1,279	363		
1367	20	454	129		
1368	20	761	216		
1369	13	405	115		
1370	3	200	57		
1371	8	356	101		
1372	16	655	186	A	
1373	18	803	228		
1374	10	451	128		
1375	13	412	117	A	
1376	24	902	256		
1377	15	821	233	A	
1378	8	433	123		
1379	8	359	102		
1380	29	1,187	337		
1381	5	338	96		
1382	7	331	94	A	
1383	6	264	75		
1384	11	486	138	A	
1385	8	419	119		
1386	7	338	96		
1387	22	705	200	D	
1388	16	740	210		
1389	26	930	264		
1390	15	666	189		
1391	9	412	117	A	
1392	21	972	276		
1393	13	648	184		
1394	4	236	67		
1395	6	190	54		
1396	5	250	71		
1397	22	831	236	A	*RDL1*
1398	7	289	82		
1399	7	246	70		
1400	14	479	136		

1401	67	2,185	620	D
1402	5	211	60	
1403	39	1,857	527	A
1404	3	162	46	
1405	3	169	48	
1406	4	165	47	
1407	54	2,333	662	A
1408	28	1,117	317	
1409	18	958	272	
1410	5	246	70	
1411	5	204	58	A
1412	13	697	198	A
1413	3	126	36	
1414	11	486	138	
1415	3	260	74	
1416	11	341	97	
1417	8	398	113	
1418	13	426	121	
1419	5	211	60	
1420	9	282	80	
1421	59	2,079	590	A
1422	3	176	50	
1423	21	786	223	A
1424	7	243	69	
1425	3	229	65	
1426	110	3,475	986	A
1427	42	1,706	484	A
1428	12	542	154	
1429	6	345	98	
1430	4	292	83	
1431	14	394	112	D
1432	4	183	52	
1433	30	1,639	465	A
1434	5	264	75	
1435	17	722	205	
1436	18	697	198	
1437	60	2,456	697	
1438	25	1,120	318	D
1439	14	348	99	
1440	3	141	40	
1441	7	299	85	
1442	4	423	120	
1443	5	296	84	
1444	19	609	173	
1445	4	267	76	A
1446	6	271	77	
1447	3	197	56	
1448	10	366	104	
1449	5	363	103	
1450	6	267	76	
1451	21	669	190	
1452	20	687	195	

1453	24	955	271	
1454	3	204	58	
1455	10	423	120	A
1456	5	412	117	
1457	15	655	186	
1458	23	817	232	A
1459	20	895	254	D
1460	4	348	99	
1461	16	511	145	
1462	15	595	169	A
1463	14	602	171	
1464	11	236	67	A+D
1465	16	419	119	A
1466	23	877	249	A
1467	5	197	56	
1468	8	394	112	
1469	9	437	124	A
1470	3	257	73	
1471	26	1,064	302	A
1472	63	2,040	579	A
1473	7	507	144	
1474	27	1,434	407	
1475	55	2,301	653	A
1476	34	1,251	355	
1477	14	482	137	
1478	4	250	71	
1479	19	669	190	
1480	8	285	81	
1481	7	278	79	
1482	6	423	120	
1483	4	289	82	A
1484	7	327	93	
1485	10	348	99	
1486	70	2,435	691	A
1487	12	705	200	A
1488	7	327	93	
1489	9	497	141	
1490	3	218	62	
1491	4	313	89	A
1492	9	380	108	
1493	12	697	198	
1494	10	437	124	
1495	28	743	211	A
1496	14	697	198	D
1497	41	1,572	446	
1498	4	267	76	
1499	8	401	114	
1500	10	437	124	A
1501	6	193	55	
1502	19	916	260	
1503	10	638	181	A
1504	34	1,508	428	

1505	32	1,177	334	A
1506	7	370	105	
1507	3	327	93	
1508	18	955	271	
1509	8	447	127	D
1510	9	525	149	
1511	39	1,304	370	A
1512	16	719	204	A
1513	4	426	121	
1514	3	193	55	
1515	7	317	90	A
1516	13	750	213	
1517	29	1,177	334	
1518	3	229	65	
1519	18	697	198	
1520	5	222	63	
1521	3	186	53	
1522	4	278	79	
1523	3	289	82	
1524	34	1,441	409	A
1525	3	179	51	
1526	5	253	72	
1527	5	338	96	A
1528	18	687	195	A
1529	5	458	130	
1530	11	567	161	
1531	5	211	60	
1532	9	394	112	A
1533	10	609	173	
1534	5	197	56	
1535	5	186	53	
1536	4	253	72	
1537	10	447	127	
1538	15	708	201	
1539	3	253	72	
1540	15	722	205	
1541	5	165	47	
1542	4	253	72	
1543	9	359	102	
1544	5	289	82	
1545	6	296	84	
1546	28	1,008	286	A
1547	5	267	76	
1548	48	1,818	516	A
1549	6	274	78	
1550	4	162	46	
1551	40	1,226	348	A
1552	19	976	277	
1553	8	398	113	
1554	17	793	225	
1555	34	1,170	332	A
1556	21	768	218	

1557	6	246	70	
1558	8	282	80	
1559	3	246	70	
1560	4	179	51	A
1561	11	408	116	
1562	103	3,232	917	D
1563	5	334	95	
1564	5	246	70	
1565	13	398	113	
1566	3	215	61	
1567	17	613	174	A
1568	25	1,004	285	A
1569	10	387	110	
1570	9	571	162	
1571	24	888	252	A
1572	42	930	264	A
1573	9	267	76	
1574	21	838	238	A
1575	10	426	121	
1576	4	218	62	A
1577	15	535	152	
1578	22	789	224	
1579	4	183	52	
1580	3	155	44	
1581	15	874	248	A
1582	4	179	51	A
1583	15	666	189	
1584	3	278	79	A
1585	10	493	140	
1586	8	433	123	A
1587	7	327	93	
1588	8	440	125	
1589	12	451	128	
1590	14	373	106	
1591	5	556	158	A
1592	17	793	225	A
1593	32	1,043	296	A
1594	6	394	112	
1595	19	757	215	
1596	5	274	78	
1597	4	324	92	
1598	32	1,061	301	A
1599	33	1,399	397	
1600	3	190	54	
1601	6	218	62	
1602	3	193	55	
1603	3	176	50	
1604	10	426	121	
1605	8	373	106	
1606	6	239	68	
1607	16	934	265	
1608	22	705	200	

1609	3	246	70	
1610	40	1,032	293	A
1611	7	225	64	
1612	3	190	54	
1613	9	433	123	
1614	4	207	59	A
1615	4	271	77	
1616	6	310	88	
1617	11	423	120	
1618	50	1,780	505	A
1619	17	458	130	
1620	64	2,347	666	A
1621	4	239	68	
1622	11	482	137	
1623	4	278	79	
1624	8	472	134	A
1625	13	853	242	
1626	14	370	105	
1627	7	282	80	
1628	24	1,078	306	A
1629	7	334	95	
1630	19	690	196	A
1631	28	1,251	355	
1632	20	705	200	
1633	7	303	86	A
1634	4	384	109	
1635	3	243	69	
1636	195	5,495	1,559	A
1637	5	433	123	
1638	5	310	88	
1639	6	225	64	A
1640	61	1,995	566	A
1641	18	835	237	A
1642	10	253	72	
1643	33	1,039	295	A
1644	10	394	112	
1645	10	296	84	
1646	3	274	78	
1647	29	934	265	A
1648	6	243	69	
1649	53	2,555	725	A
1650	38	1,445	410	A
1651	5	260	74	
1652	15	655	186	
1653	4	225	64	
1654	11	662	188	
1655	4	186	53	
1656	9	500	142	A
1657	18	761	216	
1658	17	620	176	
1659	37	1,304	370	A
1660	9	560	159	A

1661	7	296	84	A
1662	7	193	55	
1663	6	222	63	
1664	19	641	182	
1665	47	1,966	558	
1666	9	518	147	
1667	6	243	69	
1668	15	824	234	A
1669	41	1,011	287	A
1670	10	549	156	
1671	24	923	262	D
1672	8	253	72	
1673	21	687	195	
1674	5	207	59	
1675	24	1,230	349	
1676	8	391	111	
1677	4	215	61	
1678	3	172	49	A
1679	24	1,008	286	A
1680	3	176	50	
1681	27	937	266	
1682	22	1,240	352	A
1683	15	817	232	
1684	11	334	95	
1685	61	2,062	585	A
1686	17	648	184	
1687	3	253	72	
1688	12	408	116	
1689	5	401	114	A
1690	12	877	249	A
1691	4	162	46	
1692	4	303	86	
1693	6	391	111	A
1694	11	253	72	
1695	51	1,635	464	A
1696	40	1,646	467	
1697	11	549	156	A
1698	4	215	61	
1699	13	697	198	A
1700	18	874	248	
1701	26	1,290	366	A
1702	13	419	119	
1703	9	387	110	
1704	9	465	132	
1705	4	225	64	
1706	15	771	219	
1707	5	267	76	
1708	6	292	83	
1709	4	207	59	
1710	23	662	188	
1711	13	588	167	
1712	17	842	239	A

1713	26	1,131	321	
1714	16	440	125	D
1715	5	239	68	A
1716	3	176	50	
1717	6	236	67	
1718	11	574	163	
1719	5	222	63	A
1720	26	916	260	
1721	9	327	93	
1722	4	313	89	
1723	5	264	75	
1724	7	482	137	A
1725	8	465	132	
1726	5	352	100	
1727	9	468	133	
1728	6	292	83	
1729	9	461	131	
1730	5	274	78	
1731	10	271	77	A
1732	15	754	214	A
1733	4	271	77	
1734	6	324	92	
1735	10	599	170	
1736	5	274	78	A
1737	7	289	82	
1738	4	296	84	
1739	5	278	79	A
1740	5	271	77	A
1741	3	200	57	
1742	46	1,966	558	A
1743	19	782	222	
1744	46	1,963	557	A
1745	3	257	73	
1746	7	437	124	A
1747	16	895	254	
1748	4	303	86	
1749	17	546	155	A
1750	55	1,441	409	
1751	12	419	119	
1752	34	867	246	A
1753	4	215	61	
1754	21	1,011	287	
1755	11	648	184	
1756	3	119	34	
1757	16	877	249	A
1758	53	1,536	436	
1759	20	842	239	
1760	24	740	210	A
1761	6	345	98	
1762	15	641	182	A
1763	8	313	89	
1764	5	207	59	

1765	9	458	130		
1766	59	1,822	517	A	
1767	8	380	108	A	
1768	6	250	71		
1769	20	793	225		
1770	10	458	130		
1771	13	634	180		
1772	5	356	101		
1773	3	222	63		
1774	14	729	207	A	
1775	4	236	67		
1776	6	306	87		
1777	19	1,113	316	A	
1778	4	289	82		
1779	9	264	75		
1780	4	246	70		
1781	64	1,596	453	A	
1782	8	415	118	A	
1783	3	141	40		
1784	4	215	61		
1785	18	1,008	286		
1786	10	356	101		
1787	14	849	241		
1788	4	204	58		
1789	3	190	54	A	
1790	3	207	59	A	
1791	33	962	273	A	
1792	13	525	149		
1793	4	183	52		
1794	21	831	236		
1795	33	1,247	354		
1796	3	246	70		
1797	6	334	95		
1798	12	472	134		
1799	4	200	57		
1800	28	803	228	A	
1801	17	680	193	A	
1802	3	179	51		
1803	9	479	136		
1804	14	556	158		
1805	12	705	200		
1806	9	271	77	A	
1807	5	356	101		
1808	3	229	65	A	
1809	8	306	87		
1810	45	627	178	D	
1811	5	271	77		
1812	5	239	68	A	
1813	50	2,125	603	A	
1814	7	430	122		
1815	20	979	278		
1816	8	299	85		*MIC3, MIC1-15*

1817	6	200	57	
1818	57	1,868	530	A
1819	98	3,200	908	A
1820	19	705	200	
1821	56	2,227	632	A
1822	7	352	100	
1823	36	1,656	470	
1824	16	638	181	
1825	3	193	55	
1826	29	1,293	367	A
1827	5	109	31	A
1828	24	821	233	A
1829	7	405	115	
1830	11	472	134	
1831	4	253	72	A
1832	11	461	131	
1833	4	324	92	
1834	9	525	149	
1835	9	433	123	A
1836	4	190	54	
1837	61	1,899	539	A
1838	9	578	164	
1839	33	1,025	291	
1840	17	821	233	
1841	5	197	56	A
1842	3	267	76	
1843	3	267	76	
1844	5	331	94	
1845	6	423	120	
1846	4	299	85	
1847	10	564	160	A
1848	8	472	134	
1849	8	659	187	A
1850	4	200	57	A
1851	9	535	152	A
1852	27	1,001	284	
1853	6	292	83	
1854	7	243	69	
1855	6	165	47	
1856	9	352	100	
1857	4	549	156	A
1858	3	133	38	
1859	4	179	51	
1860	27	733	208	A
1861	4	158	45	
1862	7	377	107	
1863	11	736	209	A
1864	6	447	127	
1865	5	278	79	
1866	10	415	118	
1867	14	655	186	A
1868	9	630	179	A

1869	4	215	61	
1870	4	246	70	D
1871	78	2,227	632	A
1872	4	398	113	
1873	3	176	50	
1874	5	535	152	
1875	7	391	111	
1876	5	243	69	
1877	8	387	110	
1878	5	197	56	
1879	4	310	88	
1880	39	1,388	394	A
1881	33	1,233	350	
1882	30	673	191	D
1883	20	697	198	
1884	4	405	115	
1885	9	549	156	
1886	7	331	94	A
1887	8	613	174	
1888	8	377	107	
1889	26	972	276	A
1890	8	489	139	
1891	7	363	103	
1892	3	207	59	
1893	47	1,565	444	A
1894	13	461	131	A
1895	12	419	119	
1896	7	345	98	A
1897	6	207	59	
1898	9	535	152	A
1899	4	285	81	
1900	13	623	177	
1901	33	1,159	329	
1902	3	151	43	
1903	8	405	115	
1904	32	1,194	339	
1905	3	232	66	
1906	21	715	203	
1907	16	634	180	
1908	33	1,078	306	A
1909	20	377	107	
1910	5	193	55	
1911	14	687	195	
1912	4	430	122	
1913	3	338	96	A
1914	3	218	62	
1915	16	853	242	A
1916	6	204	58	
1917	11	824	234	
1918	7	324	92	A
1919	3	204	58	
1920	6	602	171	

1921	3	172	49	
1922	7	373	106	
1923	22	1,001	284	A
1924	22	867	246	A
1925	3	162	46	A
1926	23	941	267	A
1927	4	190	54	
1928	7	313	89	
1929	61	2,608	740	A
1930	8	271	77	
1931	8	246	70	A
1932	8	507	144	
1933	7	553	157	
1934	17	690	196	A
1935	7	359	102	
1936	29	1,205	342	A
1937	5	204	58	
1938	5	363	103	
1939	3	204	58	
1940	34	1,448	411	A
1941	16	733	208	A
1942	3	204	58	
1943	5	225	64	A
1944	10	430	122	
1945	24	898	255	A
1946	6	292	83	
1947	9	673	191	
1948	3	236	67	
1949	5	193	55	D
1950	8	137	39	A
1951	6	331	94	
1952	4	313	89	
1953	3	165	47	
1954	6	327	93	
1955	21	920	261	
1956	19	708	201	
1957	10	292	83	
1958	7	334	95	
1959	5	222	63	
1960	6	200	57	
1961	5	222	63	
1962	4	243	69	D
1963	21	1,106	314	A
1964	3	126	36	
1965	19	1,075	305	A
1966	7	479	136	
1967	6	415	118	
1968	14	648	184	A
1969	33	1,145	325	
1970	4	207	59	
1971	14	493	140	D
1972	21	687	195	A

1973	11	486	138	
1974	3	222	63	
1975	7	331	94	A
1976	4	179	51	
1977	5	183	52	
1978	3	215	61	
1979	4	239	68	
1980	6	433	123	
1981	14	652	185	
1982	7	380	108	
1983	3	183	52	
1984	3	225	64	
1985	13	592	168	
1986	4	285	81	
1987	22	736	209	
1988	9	423	120	A
1989	6	306	87	
1990	5	158	45	
1991	33	1,276	362	A
1992	7	415	118	A
1993	5	331	94	
1994	3	197	56	A
1995	13	553	157	A
1996	3	232	66	
1997	11	497	141	
1998	40	1,610	457	
1999	8	571	162	A
2000	15	662	188	A
2001	3	211	60	
2002	4	239	68	
2003	11	588	167	
2004	19	927	263	A
2005	4	303	86	
2006	4	193	55	
2007	5	461	131	
2008	4	218	62	
2009	15	564	160	
2010	49	1,596	453	A
2011	7	310	88	
2012	3	193	55	
2013	4	215	61	
2014	6	303	86	
2015	40	1,068	303	A
2016	7	243	69	
2017	7	310	88	
2018	25	934	265	A
2019	4	186	53	
2020	4	229	65	
2021	5	444	126	
2022	3	169	48	
2023	5	222	63	
2024	7	218	62	

2025	16	613	174	D
2026	5	306	87	
2027	5	553	157	
2028	25	987	280	A
2029	4	239	68	
2030	7	401	114	
2031	33	923	262	
2032	9	479	136	A
2033	3	197	56	
2034	16	426	121	
2035	56	1,413	401	
2036	4	193	55	A
2037	5	285	81	
2038	6	289	82	
2039	6	243	69	
2040	15	553	157	
2041	5	472	134	
2042	4	239	68	
2043	3	200	57	
2044	8	306	87	A
2045	5	222	63	
2046	15	571	162	
2047	14	898	255	
2048	5	366	104	A
2049	5	348	99	
2050	4	218	62	
2051	21	630	179	D
2052	10	454	129	
2053	8	363	103	
2054	14	828	235	A
2055	5	222	63	
2056	3	257	73	
2057	6	426	121	
2058	12	662	188	
2059	4	169	48	
2060	22	838	238	
2061	5	398	113	
2062	43	969	275	A
2063	20	641	182	
2064	8	356	101	A
2065	13	641	182	
2066	6	398	113	
2067	3	197	56	
2068	5	334	95	
2069	14	599	170	
2070	4	162	46	
2071	24	1,082	307	
2072	9	521	148	
2073	5	186	53	
2074	14	673	191	A
2075	5	465	132	
2076	3	204	58	

2077	5	260	74	
2078	6	352	100	
2079	17	694	197	
2080	4	345	98	
2081	8	451	128	
2082	40	1,522	432	A
2083	7	232	66	
2084	9	556	158	
2085	12	733	208	
2086	4	211	60	
2087	6	282	80	A
2088	15	652	185	D
2089	4	609	173	
2090	37	1,343	381	
2091	22	662	188	A
2092	6	412	117	
2093	15	669	190	
2094	9	324	92	
2095	6	292	83	
2096	7	497	141	
2097	63	1,924	546	A
2098	4	222	63	
2099	3	133	38	
2100	10	317	90	A
2101	17	726	206	A
2102	3	260	74	
2103	3	112	32	
2104	5	426	121	
2105	4	222	63	
2106	3	141	40	
2107	3	232	66	A
2108	6	285	81	
2109	4	197	56	
2110	3	232	66	
2111	26	1,367	388	
2112	11	535	152	
2113	45	1,646	467	A
2114	10	493	140	
2115	10	528	150	A
2116	13	451	128	
2117	11	676	192	
2118	3	116	33	
2119	4	186	53	
2120	12	786	223	A
2121	5	447	127	
2122	3	246	70	
2123	5	296	84	
2124	10	475	135	A
2125	12	599	170	
2126	4	292	83	
2127	10	528	150	
2128	3	204	58	

2129	67	1,871	531	A
2130	10	341	97	
2131	12	525	149	A
2132	13	574	163	
2133	5	313	89	
2134	5	264	75	
2135	7	352	100	
2136	12	535	152	
2137	25	1,392	395	
2138	6	215	61	
2139	3	200	57	
2140	3	236	67	
2141	3	193	55	
2142	13	423	120	
2143	15	465	132	
2144	29	1,099	312	
2145	17	1,043	296	A
2146	25	669	190	A
2147	26	740	210	A
2148	3	165	47	
2149	4	264	75	
2150	4	236	67	A
2151	5	437	124	
2152	4	257	73	
2153	13	645	183	
2154	31	1,089	309	A
2155	4	218	62	
2156	3	190	54	
2157	4	197	56	
2158	5	229	65	
2159	3	190	54	
2160	26	750	213	A
2161	10	482	137	A
2162	39	1,367	388	A
2163	18	867	246	
2164	25	715	203	
2165	10	556	158	A
2166	3	158	45	
2167	6	317	90	
2168	3	197	56	
2169	3	246	70	
2170	5	306	87	
2171	3	253	72	
2172	15	627	178	A
2173	4	398	113	
2174	5	313	89	A
2175	32	1,071	304	A
2176	4	222	63	
2177	18	810	230	A
2178	8	535	152	
2179	8	489	139	
2180	4	274	78	

2181	3	176	50		
2182	5	320	91		
2183	6	264	75		
2184	13	497	141	A	
2185	4	229	65		
2186	4	207	59	A	
2187	33	1,212	344	A	*CelA3, Ces*
2188	5	334	95	A	
2189	26	800	227	A	*CelA6*
2190	37	1,321	375	A	
2191	7	306	87		
2192	5	207	59		
2193	16	701	199		
2194	3	260	74		
2195	3	292	83	D	
2196	20	884	251		
2197	4	144	41		
2198	5	165	47		
2199	54	1,579	448	A	
2200	18	638	181		
2201	19	426	121		
2202	15	846	240	A	
2203	3	243	69		
2204	7	320	91	A	
2205	18	965	274		
2206	18	736	209		
2207	26	1,025	291	A	
2208	23	1,001	284	A	
2209	48	1,868	530	A	
2210	9	581	165		
2211	5	303	86		
2212	8	549	156		
2213	13	609	173		
2214	7	363	103		
2215	15	627	178		
2216	5	274	78		
2217	3	137	39		
2218	4	179	51	A	
2219	6	433	123		
2220	5	299	85	A	
2221	6	468	133	A	
2222	5	207	59		
2223	5	271	77		
2224	4	232	66		
2225	5	465	132		
2226	10	176	50		
2227	9	497	141		
2228	3	130	37		
2229	3	102	29		
2230	32	838	238	A	
2231	20	1,061	301	A	
2232	4	363	103		

2233	5	292	83	
2234	19	511	145	
2235	18	916	260	A
2236	3	158	45	
2237	3	282	80	
2238	4	229	65	
2239	18	712	202	
2240	16	902	256	
2241	5	317	90	
2242	5	363	103	D
2243	10	521	148	
2244	3	229	65	A
2245	12	638	181	
2246	3	176	50	
2247	5	412	117	
2248	7	394	112	A
2249	9	320	91	
2250	10	296	84	A
2251	24	623	177	
2252	4	232	66	A
2253	10	497	141	
2254	4	183	52	
2255	7	394	112	A
2256	12	539	153	
2257	9	401	114	
2258	7	401	114	A
2259	5	489	139	
2260	9	338	96	
2261	5	158	45	
2262	6	398	113	A
2263	4	225	64	
2264	9	666	189	
2265	6	327	93	
2266	14	750	213	A
2267	3	123	35	
2268	3	232	66	
2269	8	348	99	A
2270	5	264	75	
2271	14	549	156	
2272	22	870	247	
2273	9	581	165	
2274	4	207	59	
2275	29	803	228	D
2276	6	475	135	A
2277	8	331	94	A
2278	3	250	71	D
2279	6	373	106	A
2280	5	222	63	A
2281	13	542	154	
2282	5	366	104	
2283	31	1,410	400	A
2284	21	1,142	324	

2285	3	176	50	
2286	4	253	72	
2287	4	306	87	
2288	7	331	94	
2289	3	190	54	A
2290	8	437	124	
2291	14	613	174	
2292	7	574	163	
2293	24	1,011	287	A
2294	5	211	60	
2295	14	828	235	
2296	8	500	142	D
2297	51	1,484	421	A
2298	6	387	110	
2299	4	236	67	
2300	13	860	244	A
2301	3	193	55	
2302	16	592	168	
2303	4	285	81	A
2304	5	299	85	
2305	4	218	62	
2306	8	733	208	
2307	29	683	194	D
2308	3	267	76	
2309	22	955	271	A
2310	32	1,219	346	A
2311	15	669	190	A
2312	3	197	56	
2313	4	257	73	
2314	3	306	87	A
2315	4	190	54	
2316	20	722	205	A
2317	5	222	63	
2318	3	200	57	
2319	5	363	103	
2320	4	211	60	
2321	8	465	132	
2322	7	444	126	
2323	4	250	71	
2324	3	84	24	
2325	3	197	56	
2326	40	1,501	426	A
2327	7	317	90	
2328	3	176	50	
2329	4	348	99	
2330	9	437	124	D
2331	3	257	73	
2332	12	560	159	
2333	30	1,096	311	A
2334	5	370	105	
2335	42	1,233	350	A
2336	35	1,258	357	A

2337	52	1,343	381	A
2338	11	860	244	
2339	10	507	144	
2340	4	186	53	
2341	11	352	100	
2342	3	215	61	
2343	7	341	97	
2344	15	627	178	
2345	6	253	72	
2346	17	694	197	
2347	5	250	71	A
2348	10	243	69	A
2349	33	1,279	363	A
2350	11	444	126	
2351	34	1,424	404	A
2352	7	253	72	
2353	12	609	173	A
2354	5	366	104	
2355	9	366	104	
2356	7	225	64	
2357	6	225	64	
2358	8	588	167	A
2359	3	207	59	
2360	3	250	71	
2361	45	1,466	416	A
2362	4	186	53	
2363	11	444	126	
2364	3	172	49	
2365	48	1,092	310	A
2366	6	239	68	
2367	20	564	160	
2368	6	405	115	A
2369	24	1,191	338	A
2370	3	112	32	
2371	7	437	124	
2372	3	229	65	
2373	3	197	56	
2374	21	874	248	A
2375	8	313	89	A
2376	7	546	155	
2377	20	838	238	
2378	3	144	41	
2379	14	641	182	
2380	7	419	119	A
2381	9	430	122	
2382	5	253	72	
2383	8	229	65	
2384	5	211	60	
2385	5	334	95	
2386	3	218	62	A
2387	5	232	66	
2388	3	169	48	

2389	8	377	107	A
2390	4	218	62	
2391	3	155	44	
2392	4	352	100	
2393	8	486	138	
2394	6	257	73	
2395	7	250	71	D
2396	10	433	123	
2397	12	415	118	
2398	4	246	70	A
2399	4	200	57	
2400	3	215	61	A
2401	21	595	169	A
2402	6	542	154	
2403	15	697	198	
2404	4	126	36	
2405	7	296	84	
2406	3	225	64	
2407	7	299	85	
2408	10	310	88	
2409	9	574	163	
2410	4	211	60	
2411	10	222	63	A
2412	7	468	133	
2413	14	507	144	
2414	21	1,173	333	
2415	5	282	80	
2416	10	525	149	
2417	3	186	53	
2418	8	546	155	
2419	49	1,688	479	A
2420	6	380	108	
2421	3	215	61	D
2422	7	348	99	
2423	8	412	117	D
2424	8	423	120	
2425	10	623	177	A
2426	3	250	71	
2427	9	521	148	
2428	10	401	114	
2429	24	775	220	A
2430	4	296	84	A
2431	16	356	101	
2432	4	197	56	
2433	7	320	91	
2434	7	345	98	A
2435	3	88	25	
2436	3	165	47	
2437	3	109	31	
2438	3	123	35	
2439	20	680	193	A
2440	3	215	61	

2441	9	676	192	
2442	28	944	268	
2443	3	158	45	
2444	4	239	68	
2445	3	200	57	
2446	21	920	261	A
2447	5	532	151	
2448	5	348	99	
2449	5	200	57	
2450	28	958	272	A
2451	4	296	84	
2452	3	236	67	A
2453	22	1,145	325	
2454	3	179	51	
2455	6	338	96	A
2456	7	415	118	
2457	4	331	94	
2458	5	186	53	
2459	7	609	173	A
2460	17	920	261	
2461	47	1,543	438	A
2462	62	1,871	531	A
2463	3	246	70	
2464	5	215	61	A
2465	34	1,328	377	A
2466	4	250	71	
2467	8	454	129	
2468	8	387	110	
2469	13	271	77	D
2470	4	352	100	A
2471	8	461	131	
2472	17	408	116	D
2473	4	225	64	
2474	3	183	52	
2475	12	690	196	
2476	9	454	129	A
2477	8	253	72	A
2478	4	211	60	A
2479	6	317	90	
2480	3	179	51	A
2481	4	373	106	
2482	9	750	213	A
2483	3	317	90	
2484	11	535	152	
2485	19	838	238	
2486	51	1,804	512	
2487	3	186	53	
2488	15	602	171	
2489	9	419	119	
2490	6	398	113	
2491	3	165	47	
2492	4	278	79	A

2493	9	451	128	
2494	3	274	78	
2495	8	574	163	
2496	19	440	125	
2497	3	232	66	
2498	4	236	67	A
2499	14	553	157	
2500	6	447	127	
2501	4	257	73	
2502	16	1,050	298	A
2503	5	232	66	
2504	9	398	113	
2505	4	412	117	
2506	24	771	219	
2507	3	299	85	
2508	5	398	113	
2509	4	144	41	
2510	26	722	205	A
2511	4	207	59	
2512	3	204	58	A
2513	3	250	71	A
2514	9	437	124	
2515	3	172	49	
2516	4	239	68	
2517	3	299	85	
2518	3	200	57	
2519	44	627	178	
2520	22	676	192	D
2521	4	193	55	
2522	5	377	107	A
2523	11	313	89	
2524	10	239	68	
2525	3	155	44	
2526	39	1,216	345	A
2527	9	426	121	A
2528	3	236	67	
2529	8	398	113	
2530	3	225	64	
2531	3	207	59	
2532	13	525	149	D
2533	3	186	53	A
2534	3	172	49	
2535	3	144	41	
2536	3	225	64	
2537	7	507	144	A
2538	3	162	46	
2539	3	193	55	
2540	5	236	67	
2541	5	282	80	
2542	5	292	83	A
2543	3	155	44	
2544	4	306	87	A

2545	3	260	74		
2546	3	148	42		
2547	4	278	79	A	
2548	5	352	100		
2549	4	250	71		
2550	9	320	91		
2551	11	800	227		
2552	21	585	166		
2553	3	264	75		
2554	3	232	66		
2555	7	405	115		
2556	5	278	79		
2557	13	648	184		
2558	4	246	70		
2559	5	236	67		
2560	3	282	80		
2561	5	313	89		
2562	38	1,392	395	A	
2563	10	511	145		
2564	4	215	61		
2565	13	525	149		
2566	39	1,617	459	A	
2567	3	172	49		
2568	3	186	53		
2569	4	207	59		
2570	3	169	48		
2571	5	310	88		
2572	13	486	138		
2573	7	366	104	A	
2574	3	204	58		
2575	3	169	48		
2576	15	437	124		
2577	27	1,367	388	A	
2578	5	264	75		
2579	8	564	160	A	
2580	12	655	186	A	
2581	5	331	94		
2582	3	215	61	A	
2583	5	225	64	A	*MIC3, NIC1-15*
2584	6	419	119		
2585	6	267	76		
2586	7	338	96		
2587	12	320	91		
2588	3	299	85		
2589	4	239	68		
2590	3	225	64		
2591	3	271	77		
2592	28	881	250		
2593	5	310	88		
2594	4	243	69		
2595	4	229	65		
2596	17	592	168	A	

2597	3	250	71	
2598	11	726	206	A
2599	3	176	50	
2600	103	3,725	1,057	A
2601	3	250	71	
2602	3	144	41	
2603	3	250	71	
2604	3	299	85	
2605	5	215	61	
2606	3	204	58	
2607	14	705	200	
2608	5	289	82	
2609	6	331	94	
2610	4	232	66	
2611	24	1,103	313	A
2612	3	204	58	
2613	6	232	66	
2614	3	165	47	
2615	4	165	47	
2616	12	627	178	
2617	5	345	98	
2618	32	613	174	A
2619	4	366	104	A
2620	3	218	62	A
2621	3	116	33	
2622	3	253	72	
2623	3	148	42	
2624	17	542	154	A
2625	3	162	46	
2626	4	253	72	A
2627	4	193	55	A
2628	4	327	93	
2629	4	303	86	
2630	12	683	194	
2631	4	119	34	
2632	4	243	69	
2633	4	546	155	A
2634	4	331	94	
2635	3	236	67	A
2636	6	271	77	
2637	10	497	141	A
2638	6	370	105	
2639	3	296	84	
2640	4	253	72	A
2641	3	222	63	
2642	6	296	84	
2643	18	789	224	
2644	13	567	161	
2645	9	327	93	
2646	5	204	58	
2647	5	229	65	A
2648	8	564	160	A

2649	44	1,223	347	
2650	3	222	63	A
2651	12	299	85	
2652	4	211	60	
2653	7	779	221	
2654	72	2,266	643	A
2655	14	697	198	
2656	44	1,286	365	A
2657	3	218	62	
2658	6	324	92	
2659	3	162	46	A
2660	6	352	100	
2661	45	1,625	461	
2662	3	246	70	
2663	5	257	73	
2664	3	197	56	
2665	47	1,378	391	A
2666	9	574	163	A
2667	3	179	51	
2668	3	186	53	
2669	17	726	206	A
2670	8	384	109	
2671	13	634	180	
2672	3	222	63	
2673	3	229	65	A
2674	4	204	58	
2675	3	172	49	
2676	5	317	90	
2677	3	334	95	
2678	3	200	57	
2679	3	267	76	
2680	5	292	83	
2681	3	197	56	
2682	3	222	63	
2683	4	207	59	
2684	15	747	212	D
2685	4	345	98	
2686	3	172	49	
2687	7	447	127	
2688	3	229	65	
2689	15	546	155	A
2690	7	535	152	
2691	3	162	46	
2692	8	299	85	
2693	4	373	106	
2694	6	267	76	
2695	3	190	54	
2696	3	151	43	A
2697	4	239	68	
2698	3	225	64	
2699	6	398	113	
2700	5	331	94	

2701	3	144	41	A	
2702	3	197	56		
2703	3	215	61		
2704	6	377	107		
2705	3	176	50		
2706	6	489	139	A	
2707	3	218	62		
2708	3	282	80		
2709	3	215	61		
2710	7	433	123		
2711	13	645	183		
2712	3	211	60		
2713	4	172	49		
2714	3	172	49		
2715	4	190	54		
2716	3	271	77		*MIC*
2717	4	289	82		
2718	7	447	127		
2719	10	715	203		
2720	20	807	229	A	
2721	6	257	73	D	
2722	3	155	44		
2723	22	874	248	A	*MIC5, MIC1-15, MYBT2*
2724	7	394	112	A	
2725	6	356	101	A	
2726	12	338	96	A	
2727	4	155	44	D	
2728	6	380	108		
2729	3	193	55		
2730	17	726	206		
2731	4	296	84		
2732	23	775	220	A	
2733	8	641	182		
2734	5	257	73		
2735	3	172	49		
2736	4	331	94		
2737	4	260	74		
2738	9	613	174	A	
2739	4	243	69	D	
2740	4	391	111	A	
2741	11	380	108		
2742	3	197	56		
2743	4	415	118		
2744	3	204	58		
2745	18	627	178	D	
2746	4	190	54		
2747	7	412	117	A	
2748	6	250	71		
2749	3	197	56	D	
2750	3	243	69		
2751	5	405	115		
2752	24	912	259	A	

2753	3	296	84	
2754	4	253	72	
2755	6	317	90	
2756	18	909	258	A
2757	43	1,466	416	A
2758	5	373	106	D
2759	6	324	92	A
2760	3	225	64	
2761	7	292	83	
2762	3	176	50	
2763	4	211	60	
2764	4	218	62	
2765	48	1,159	329	D
2766	3	190	54	
2767	12	743	211	
2768	3	211	60	A
2769	6	200	57	
2770	10	775	220	
2771	13	331	94	A
2772	10	567	161	
2773	6	366	104	A
2774	4	232	66	
2775	3	306	87	
2776	3	172	49	A
2777	30	1,346	382	A
2778	6	556	158	
2779	5	292	83	
2780	3	200	57	A
2781	3	232	66	
2782	3	260	74	A
2783	6	278	79	A
2784	4	257	73	A
2785	6	215	61	D
2786	4	289	82	A
2787	17	821	233	A
2788	4	236	67	
2789	7	324	92	
2790	6	320	91	A
2791	34	891	253	A
2792	5	472	134	
2793	13	613	174	A
2794	8	183	52	
2795	34	1,008	286	A
2796	6	303	86	
2797	3	172	49	
2798	60	2,167	615	A
2799	9	391	111	
2800	13	461	131	A
2801	41	835	237	A
2802	10	542	154	
2803	9	458	130	
2804	3	246	70	

2805	4	303	86	A
2806	13	384	109	A
2807	3	183	52	
2808	3	190	54	
2809	14	793	225	A
2810	4	289	82	A
2811	3	190	54	
2812	5	253	72	A
2813	3	200	57	
2814	3	172	49	
2815	10	341	97	
2816	6	528	150	
2817	6	408	116	A
2818	3	225	64	
2819	3	232	66	
2820	8	243	69	
2821	4	331	94	
2822	4	204	58	
2823	3	236	67	
2824	20	743	211	
2825	6	222	63	
2826	4	186	53	
2827	13	482	137	
2828	7	398	113	A
2829	11	556	158	
2830	3	204	58	
2831	4	274	78	
2832	22	874	248	A
2833	3	176	50	A
2834	3	211	60	
2835	4	430	122	
2836	3	207	59	
2837	5	207	59	
2838	3	155	44	
2839	28	983	279	
2840	5	401	114	
2841	3	260	74	
2842	12	768	218	
2843	3	162	46	A
2844	4	253	72	
2845	3	179	51	
2846	15	433	123	A
2847	3	313	89	
2848	14	726	206	
2849	3	200	57	
2850	18	712	202	
2851	21	726	206	
2852	3	215	61	
2853	3	197	56	A
2854	8	454	129	
2855	15	796	226	
2856	5	197	56	

2857	3	246	70	
2858	56	1,776	504	A
2859	3	207	59	
2860	4	204	58	A
2861	3	211	60	A
2862	4	246	70	
2863	3	264	75	
2864	4	225	64	
2865	3	197	56	
2866	3	197	56	
2867	3	239	68	
2868	5	197	56	A
2869	7	282	80	
2870	4	239	68	
2871	11	518	147	
2872	3	197	56	
2873	3	204	58	
2874	12	479	136	
2875	3	148	42	
2876	8	391	111	
2877	5	193	55	A
2878	3	204	58	
2879	3	211	60	
2880	6	239	68	
2881	14	853	242	
2882	5	296	84	
2883	12	849	241	
2884	3	239	68	
2885	5	299	85	
2886	6	299	85	
2887	3	207	59	
2888	4	380	108	
2889	4	401	114	
2890	3	179	51	
2891	5	236	67	
2892	8	768	218	
2893	9	493	140	A
2894	7	444	126	
2895	3	264	75	
2896	10	599	170	
2897	6	581	165	A
2898	4	306	87	A
2899	6	440	125	
2900	3	200	57	A
2901	6	253	72	A
2902	3	232	66	
2903	14	662	188	
2904	3	204	58	A
2905	3	179	51	
2906	18	690	196	
2907	7	370	105	
2908	6	535	152	

2909	5	267	76	
2910	4	373	106	
2911	4	271	77	
2912	3	169	48	
2913	3	271	77	
2914	4	218	62	
2915	4	444	126	
2916	3	176	50	
2917	3	183	52	
2918	3	215	61	
2919	3	222	63	
2920	3	155	44	A
2921	35	930	264	
2922	7	472	134	
2923	3	172	49	
2924	9	549	156	A
2925	16	588	167	
2926	9	423	120	A
2927	4	384	109	
2928	20	708	201	D
2929	3	218	62	
2930	5	282	80	A
2931	7	324	92	A
2932	4	415	118	
2933	10	440	125	
2934	9	320	91	A
2935	9	546	155	
2936	48	1,455	413	A
2937	16	461	131	
2938	7	423	120	
2939	3	193	55	
2940	3	183	52	
2941	4	197	56	
2942	18	846	240	A
2943	5	271	77	A
2944	4	489	139	
2945	9	564	160	A
2946	4	366	104	
2947	17	687	195	A
2948	3	130	37	
2949	3	207	59	
2950	8	489	139	
2951	6	507	144	A
2952	3	179	51	
2953	32	645	183	A
2954	4	183	52	A
2955	12	472	134	
2956	41	1,780	505	
2957	7	310	88	
2958	4	151	43	A
2959	3	229	65	A
2960	3	250	71	

2961	4	310	88	A
2962	3	144	41	
2963	15	719	204	
2964	6	391	111	
2965	3	222	63	
2966	23	874	248	
2967	4	465	132	
2968	3	165	47	
2969	3	193	55	
2970	59	1,787	507	A
2971	16	613	174	
2972	23	750	213	
2973	3	197	56	
2974	3	186	53	
2975	3	183	52	
2976	17	687	195	
2977	6	426	121	
2978	11	451	128	A
2979	5	218	62	
2980	11	479	136	
2981	3	239	68	
2982	8	260	74	
2983	8	507	144	
2984	3	133	38	D
2985	11	232	66	
2986	6	236	67	
2987	3	176	50	
2988	3	158	45	
2989	9	444	126	
2990	19	1,011	287	
2991	9	285	81	
2992	3	218	62	
2993	3	158	45	
2994	17	465	132	A
2995	3	126	36	
2996	4	169	48	
2997	5	320	91	
2998	4	285	81	
2999	3	176	50	
3000	5	257	73	D
3001	5	215	61	
3002	14	401	114	
3003	17	267	76	A
3004	25	486	138	
3005	26	669	190	
3006	16	373	106	
3007	23	927	263	
3008	35	764	217	D
3009	47	1,522	432	A
3010	5	317	90	
3011	8	377	107	
3012	26	962	273	A

3013	7	447	127	D
3014	10	468	133	
3015	18	810	230	
3016	21	877	249	A
3017	13	521	148	
3018	55	1,519	431	
3019	6	200	57	
3020	17	764	217	
3021	22	729	207	D
3022	23	743	211	D
3023	10	398	113	
3024	6	352	100	
3025	34	1,106	314	A
3026	28	1,064	302	A
3027	10	592	168	A
3028	8	334	95	
3029	7	324	92	
3030	33	652	185	
3031	17	683	194	
3032	3	253	72	
3033	3	183	52	
3034	10	475	135	
3035	38	1,223	347	A
3036	37	648	184	A
3037	9	341	97	
3038	16	426	121	A
3039	17	733	208	A
3040	17	574	163	
3041	4	193	55	
3042	13	521	148	A
3043	3	179	51	
3044	5	239	68	
3045	4	267	76	
3046	3	232	66	
3047	6	246	70	
3048	6	299	85	A
3049	22	937	266	A
3050	13	447	127	D
3051	3	317	90	A
3052	7	285	81	
3053	13	511	145	A
3054	16	662	188	
3055	14	472	134	
3056	7	285	81	
3057	4	218	62	
3058	3	264	75	
3059	3	141	40	
3060	4	211	60	
3061	12	528	150	
3062	14	694	197	A
3063	11	497	141	A
3064	17	645	183	

3065	8	274	78	D
3066	16	433	123	
3067	6	162	46	
3068	22	444	126	
3069	6	260	74	
3070	15	518	147	
3071	6	317	90	
3072	11	373	106	A
3073	3	172	49	
3074	10	285	81	
3075	10	451	128	
3076	13	225	64	
3077	3	207	59	
3078	6	324	92	
3079	8	250	71	
3080	7	423	120	A
3081	5	267	76	A
3082	5	264	75	
3083	8	338	96	
3084	10	239	68	
3085	20	356	101	
3086	3	186	53	
3087	5	250	71	
3088	6	271	77	
3089	30	860	244	
3090	9	313	89	A
3091	4	172	49	
3092	6	232	66	
3093	6	179	51	
3094	11	236	67	
3095	3	165	47	A
3096	10	345	98	
3097	13	253	72	
3098	6	179	51	
3099	3	186	53	
3100	3	222	63	
3101	3	296	84	A
3102	13	588	167	A
3103	4	282	80	
3104	4	222	63	
3105	3	264	75	
3106	10	334	95	
3107	3	250	71	
3108	7	327	93	A
3109	3	172	49	
3110	3	186	53	
3111	6	437	124	A
3112	21	789	224	
3113	4	204	58	
3114	4	197	56	
3115	10	415	118	
3116	3	356	101	A

3117	5	148	42	
3118	14	497	141	D
3119	14	546	155	
3120	6	172	49	
3121	4	200	57	
3122	6	193	55	
3123	3	183	52	
3124	9	479	136	
3125	5	158	45	
3126	4	264	75	
3127	4	236	67	
3128	4	271	77	
3129	4	239	68	
3130	5	352	100	
3131	11	320	91	D
3132	13	384	109	
3133	5	179	51	
3134	3	278	79	
3135	6	207	59	
3136	9	405	115	
3137	4	243	69	
3138	5	169	48	
3139	8	331	94	
3140	6	236	67	
3141	11	373	106	
3142	6	236	67	
3143	6	200	57	
3144	11	327	93	
3145	11	267	76	
3146	12	380	108	
3147	9	260	74	
3148	9	218	62	
3149	8	377	107	
3150	19	475	135	
3151	11	229	65	
3152	12	437	124	A
3153	8	278	79	
3154	45	888	252	A
3155	11	289	82	A
3156	7	239	68	
3157	8	289	82	
3158	12	511	145	A
3159	12	539	153	A
3160	12	482	137	
3161	9	458	130	A
3162	4	197	56	A
3163	24	528	150	
3164	17	549	156	
3165	11	394	112	
3166	6	190	54	D
3167	12	278	79	
3168	12	426	121	

3169	12	331	94	
3170	3	130	37	D
3171	3	186	53	A+D
3172	3	225	64	A
3173	30	944	268	A
3174	13	497	141	A
3175	11	232	66	
3176	5	370	105	
3177	4	274	78	A
3178	4	229	65	
3179	5	207	59	D
3180	13	507	144	
3181	4	186	53	A
3182	8	183	52	
3183	4	179	51	
3184	10	225	64	
3185	7	303	86	
3186	3	158	45	
3187	4	243	69	
3188	7	356	101	
3189	7	391	111	
3190	6	250	71	A
3191	5	193	55	
3192	6	313	89	
3193	4	257	73	
3194	5	289	82	
3195	3	197	56	
3196	5	232	66	
3197	4	289	82	
3198	7	345	98	
3199	9	303	86	
3200	18	616	175	
3201	6	348	99	
3202	9	292	83	
3203	8	204	58	A
3204	3	274	78	A
3205	7	401	114	A
3206	4	162	46	
3207	7	193	55	
3208	6	299	85	
3209	8	489	139	
3210	6	232	66	
3211	7	320	91	
3212	12	317	90	
3213	18	426	121	D
3214	11	317	90	
3215	7	317	90	
3216	3	193	55	
3217	5	246	70	A
3218	8	387	110	
3219	7	299	85	
3220	14	475	135	D

3221	33	451	128	A	
3222	7	345	98	D	
3223	8	419	119	D	
3224	5	137	39		
3225	22	807	229	A	
3226	49	571	162	D	
3227	75	2,055	583	A	
3228	6	200	57		
3229	13	408	116	D	
3230	3	148	42		
3231	4	193	55		
3232	8	320	91	A	
3233	7	109	31	D	
3234	5	222	63		
3235	19	514	146	A	
3236	4	246	70		
3237	28	796	226	A	
3238	3	155	44	A	
3239	8	236	67	D	
3240	13	387	110	A	
3241	7	222	63		
3242	10	345	98	A	
3243	26	722	205	D	
3244	4	197	56		
3245	6	260	74		
3246	6	310	88	A	
3247	41	831	236	A	*MYBT2*
3248	4	211	60		
3249	9	299	85	A	
3250	3	158	45		
3251	4	207	59		
3252	12	331	94		
3253	24	532	151	A	
3254	33	814	231	A	
3255	6	303	86	A	
3256	4	211	60	A	
3257	10	296	84		
3258	6	250	71		
3259	7	186	53		
3260	7	394	112	D	
3261	11	317	90	A	
3262	13	285	81	A	
3263	23	697	198	A	
3264	11	303	86		
3265	3	190	54		
3266	3	211	60	D	
3267	6	299	85		
3268	30	708	201	A	
3269	16	482	137	A	
3270	11	401	114		
3271	3	155	44		
3272	3	289	82	D	

3273	15	207	59	A
3274	6	324	92	
3275	3	172	49	
3276	5	169	48	
3277	10	292	83	
3278	7	341	97	
3279	8	352	100	
3280	33	645	183	A
3281	10	352	100	
3282	7	292	83	
3283	14	363	103	A
3284	22	796	226	
3285	12	236	67	
3286	25	609	173	D
3287	13	331	94	
3288	6	292	83	
3289	18	511	145	A
3290	3	215	61	A
3291	6	141	40	
3292	9	264	75	
3293	9	373	106	A
3294	4	172	49	
3295	15	518	147	A
3296	5	338	96	
3297	6	239	68	D
3298	12	553	157	
3299	29	553	157	D
3300	21	627	178	A
3301	15	352	100	A
3302	4	211	60	A
3303	10	204	58	
3304	19	585	166	A
3305	14	359	102	D
3306	12	384	109	A
3307	10	345	98	A
3308	12	229	65	
3309	8	370	105	A
3310	9	179	51	A
3311	10	338	96	
3312	11	352	100	D
3313	9	190	54	
3314	5	299	85	
3315	4	232	66	
3316	7	264	75	
3317	5	274	78	
3318	18	437	124	D
3319	15	433	123	D
3320	6	267	76	
3321	37	1,202	341	A
3322	17	348	99	A
3323	12	211	60	
3324	8	207	59	A

3325	8	296	84	A
3326	8	313	89	A
3327	20	574	163	D
3328	17	486	138	A
3329	25	475	135	D
3330	46	860	244	A
3331	4	200	57	
3332	9	246	70	
3333	16	602	171	
3334	6	176	50	D
3335	35	768	218	A
3336	4	172	49	A
3337	4	176	50	A
3338	27	313	89	D
3339	5	204	58	
3340	6	274	78	
3341	34	461	131	D
3342	3	197	56	
3343	10	165	47	
3344	13	324	92	A
3345	10	267	76	D
3346	50	1,202	341	A
3347	10	535	152	D
3348	15	454	129	A
3349	4	169	48	
3350	3	130	37	
3351	4	162	46	
3352	4	176	50	
3353	10	398	113	A
3354	7	303	86	
3355	6	193	55	
3356	4	172	49	A
3357	7	296	84	
3358	10	313	89	A
3359	7	193	55	
3360	5	225	64	
3361	8	119	34	
3362	11	218	62	D
3363	13	292	83	A
3364	5	296	84	
3365	8	267	76	A
3366	23	585	166	D
3367	9	398	113	
3368	14	623	177	
3369	9	267	76	
3370	3	207	59	A
3371	6	222	63	
3372	7	341	97	
3373	25	458	130	A+D
3374	5	289	82	
3375	19	447	127	A
3376	4	320	91	

3377	7	278	79	D	
3378	11	299	85	A	
3379	4	112	32		
3380	9	405	115	A	
3381	13	292	83		
3382	19	331	94	D	
3383	5	246	70		
3384	39	1,209	343	A	
3385	3	162	46	D	
3386	13	257	73		
3387	4	141	40		
3388	7	299	85	D	
3389	3	186	53		
3390	9	204	58	A	
3391	5	207	59		
3392	4	109	31	A	
3393	9	239	68	A	
3394	6	158	45		
3395	5	148	42		
3396	7	204	58		
3397	3	109	31		
3398	10	257	73		
3399	5	253	72		
3400	4	218	62	A	
3401	7	218	62		
3402	15	215	61		
3403	8	126	36	A	
3404	6	123	35	A	
3405	3	158	45	A	
3406	10	162	46		
3407	11	331	94		
3408	8	218	62		
3409	8	296	84		
3410	5	176	50		
3411	4	183	52		
3412	5	215	61		
3413	3	229	65		
3414	14	165	47		
3415	4	158	45		
3416	9	345	98		
3417	4	169	48		
3418	51	567	161	A	
3419	6	109	31		
3420	7	207	59		
3421	16	292	83		
3422	6	200	57		
3423	3,683	1,614	458	A+D	*GhCesA2*
3424	6	179	51		
3425	4	186	53		
3426	282	923	262	D	
3427	3	218	62		
3428	94	405	115	A	

3429	8	211	60	
3430	8	123	35	
3431	6	84	24	
3432	15	253	72	
3433	6	158	45	
3434	6	239	68	
3435	8	253	72	
3436	7	193	55	
3437	24	433	123	D
3438	10	225	64	D
3439	6	200	57	
3440	9	310	88	
3441	11	426	121	
3442	19	489	139	
3443	5	320	91	
3444	12	267	76	A
3445	17	363	103	
3446	8	162	46	
3447	7	257	73	
3448	7	193	55	
3449	10	479	136	A
3450	3	119	34	
