# Supplementary material for: Genome Physical Mapping of Polyploids: A BIBAC Physical Map of Cultivated Tetraploid Cotton, Gossypium hirsutum L
Source: PLoS One. 2012 Mar 16;7(3):e33644. doi: 10.1371/journal.pone.0033644 (PMC3306275; doi:10.1371/journal.pone.0033644)
Supplement: Table S3 — Positive clones of A-subgenome specific probes, pXP128 and pXP137, and D-subgenome specific probe, pXP195. (PDF) [file pone.0033644.s004.pdf]

Table S3. Positive clones of A-subgenome specific probes, pXP128 and pXP137, and D-subgenome specific probe, pXP195.

pXP128	pXP137	pXP195
B003A13	B002A02	B008A06
B006A14	B007A04	B002A18
B004A23	B005A05	B004B05
B005B08	B004A23	B007B19
B003B16	B002B01	B006B24
B001B18	B002B02	B005A05
B002C01	B006B03	B001D04
B005C20	B001B10	B008D04
B008D04	B003B16	B002D06
B007E04	B001B18	B004D08
B004E07	B002C01	B006C03
B002E09	B005C02	B001C14
B008E11	B006C03	B004C18
B008D14	B007C10	B006D15
B001E12	B001C18	B002E15
B001E15	B004C22	B003E21
B008F04	B001D02	B006E17
B007F24	B001D04	B006E23
B003G02	B008D04	B006F03
B008G03	B001D05	B005F12
B001G05	B007E04	B007E04
B008G09	B003E13	B002E08
B004G10	B008F01	B007F03
B006g11	B001F03	B008G03
B001H02	B007F03	B006G05
B008H04	B003G02	B008H04
B001H07	B008G03	B001H07
B001H08	B001H02	B005G03
B002H13	B008H04	B005G06
B003I01	B001H07	B001G23
B005I02	B001K06	B005G24
B008J03	B008K06	B005H01
B006J12	B005K24	B008H05
B003K13	B002L01	B005I03
B005K24	B001L02	B005J01
B005L01	B008L02	B008J20
B005L07	B005L07	B008J22
B002L09	B003L18	B001I04
B003L11	B008L23	B008J03
B008L23	B004M02	B001K04
B007M03	B007M03	B002L09
B002M09	B002M09	B004K05
B004M13	B003M13	B008L06
B007M16	B004M13	B008L23
B005N02	B003M14	B002L24
B008N04	B007M16	B004M10
B001N11	B002N02	B002M17

B007N23	B008N04	B006N08
B006N15	B005N05	B007N22
B006O02	B005N06	B004N23
B004O07	B003N21	B002M09
B002O20	B007N23	B002N02
B001P04	B006O02	B008N03
B007P13	B007O06	B001N11
B004P16	B004O07	B004O07
B003P21	B004O14	B002O20
B005A05	B002O20	B002P02
B004C22	B003O21	B004P03
B002D24	B005O22	B001P04
B006F09	B002P02	B002P08
B007F17	B004P03	B007P13
B004I17	B001P04	B003O18
B007J17	B008P04	B008P21
B003L18	B004P16	B002P22
B003M14	B007P18	B017A01
B002N08	B003P21	B019B03
B005O22	B021A01	B017B07
B007P01	B018A04	B017D13
B002P02	B023A04	B022D16
B004P03	B023A07	B013A04
B021A01	B021A08	B023A07
B023A07	B022A14	B017A12
B021A08	B021A20	B022A14
B022A14	B018A22	B023B02
B022A15	B023B01	B023B08
B018A22	B023B02	B020B21
B023B01	B021B04	B020C22
B023B08	B023B08	B023D12
B024C04	B020B21	B024E03
B020C09	B022B23	B021E08
B019C10	B024C04	B018E11
B020C20	B020C09	B018E22
B021C21	B023C12	B024G02
B020C22	B020C22	B024G05
B020C23	B020C23	B024E04
B022B23	B024C24	B019F13
B019D03	B020D01	B019F18
B023D12	B017D04	B024F13
B020D13	B020D09	B023H11
B024E03	B017D23	B024I03
B022E09	B018E01	B018J18
B021E13	B024E03	B021J24
B018F13	B018E22	B017K02
B017F14	B021F24	B023K22
B020F12	B024G02	B019L05
B021F24	B024G03	B023L09
B024G02	B022G15	B018J07
B021H01	B021G23	B023K12
B017H13	B023H14	B018O01

B023H14	B021H17	B017O02
B021H19	B021H19	B020O04
B023I11	B022I15	B018O09
B023I14	B020K02	B018O18
B018J07	B017K23	B018P01
B021J13	B020L02	B020P02
B024J20	B023M05	B017P04
B020K05	B020M18	B017O17
B024K08	B023M21	B020O21
B023K12	B021M23	B017M23
B020L05	B024N02	B020O19
B017L07	B019N03	B022P04
B019L17	B017N04	B022P05
B018L21	B022N05	B016B14
B019M05	B019N11	B014B16
B019M06	B020N17	B009C01
B019M09	B018O01	B013B04
B021M23	B017O02	B011B06
B019N03	B020O04	B010B11
B019N04	B021O04	B015B13
B022N05	B020O05	B012B20
B019N10	B018O09	B011D02
B019N11	B018O12	B013D08
B020N14	B017O14	B010A20
B020N17	B018O18	B010A22
B020N18	B018O20	B013B01
B018O01	B018P01	B015B09
B017O02	B020P02	B016B13
B018O09	B018P03	B016B21
B020P02	B017P04	B012C03
B017P04	B023P04	B010C04
B019P10	B021P06	B014C07
B020P15	B022P06	B012D04
B024P16	B019P08	B009E01
B017P17	B019P10	B011F13
B020P21	B018P14	B016H07
B020A04	B024P16	B016E02
B012A20	B017P17	B013E10
B020B06	B020P21	B012E14
B024C06	B021P22	B009F07
B019C16	B040A01	B015H13
B020D05	B033A02	B013H15
B017D18	B036A05	B015I15
B023F08	B036A06	B013I17
B018F11	B035A13	B010J22
B020F15	B034A14	B010K24
B017F17	B033A15	B015L08
B024G05	B035A18	B011I19
B019G13	B037A21	B015L02
B022G15	B035A22	B015L21
B020H10	B040A23	B011L24
B020I06	B036B03	B009M09

B018I13	B033B05	B015N01
B022I15	B037B08	B009P14
B020L02	B033B12	B012P22
B018L09	B039B12	B010M08
B019M10	B040B13	B009M13
B020M18	B040B15	B012M16
B023M21	B036B16	B016N03
B024N02	B036B17	B014N10
B018O18	B040B20	B014O07
B023O21	B035C03	B011O09
B018P14	B034C05	B013O19
B036A06	B036C09	B011O21
B034A14	B035C12	B012O23
B033A15	B040C17	B038A04
B035A18	B033C18	B034A18
B037A21	B034C20	B039A18
B035A22	B036C20	B039A19
B040A23	B035D15	B040B02
B039B01	B033D24	B035B03
B033B05	B035E01	B035B16
B035B07	B036E18	B040D14
B040B13	B035F01	B040D24
B040B14	B040F04	B033A02
B040B15	B038F07	B034C20
B033B16	B038F08	B039D01
B036B24	B037F15	B033D24
B034C05	B034F16	B039F08
B036C09	B036F16	B034G03
B037C14	B036G01	B033G20
B040C17	B035G02	B034G22
B033C18	B034G22	B034G23
B034C20	B034G23	B040E12
B034C24	B038H21	B034F20
B040D06	B033I02	B033G19
B037D21	B036I17	B038G20
B033D24	B035I20	B040H01
B035E01	B035I22	B036H06
B036E03	B034J06	B039I21
B036E05	B035J08	B036J14
B040E07	B033J16	B037K21
B034E09	B037J17	B036L11
B037E13	B033J18	B034L15
B039E22	B033K02	B034J22
B035F01	B034K03	B038J02
B040F04	B034K04	B034K02
B039F08	B039K13	B040M10
B037F12	B039K17	B036M20
B033F14	B040K21	B040N05
B037F15	B035L09	B033N06
B034F16	B037L12	B037N08
B035G02	B035M01	B033N20
B033G05	B038M04	B036N23

B036G14	B040M04	B038O04
B034G23	B036M09	B036O21
B040H01	B036M11	B034P18
B038H21	B036M20	B036P17
B035H24	B035M24	B039P20
B033I02	B038N02	B037P21
B039I08	B034N02	B033P22
B035I22	B033N06	B034O07
B037I24	B034N09	B040O10
B039J01	B038N20	B036O22
B035J08	B038N24	B040N08
B034J10	B038O02	B038N12
B038J14	B038O04	B033N14
B034J22	B038O05	B033N18
B039K13	B034O19	B025A03
B040K21	B035O19	B030A05
B039L07	B036O21	B025B07
B037L12	B038P07	B031B07
B040M04	B036P08	B030B12
B036M11	B036P09	B029B15
B035M24	B034P18	B028B23
B037N08	B038P20	B025C05
B036P11	B034P21	B025C07
B033A02	B035P23	B032C14
B038B06	B037P23	B026A12
B040B11	B012A02	B028B02
B033B12	B015A06	B031B18
B039B20	B010A20	B026C23
B040B22	B010A22	B025D21
B033C08	B011B02	B032E02
B035C12	B009B04	B032F02
B040D04	B013B05	B028F04
B036E20	B016B21	B028H13
B038F24	B010B23	B025H22
B036H09	B014B24	B025E07
B038H10	B016C01	B025E08
B037H12	B012C03	B029E10
B037H19	B010C04	B031E19
B036I10	B014D03	B028F04
B036I16	B012D04	B026F05
B036I17	B012E20	B031F05
B037I21	B016E21	B028F09
B037J15	B014F03	B032F10
B033J16	B009F05	B032G02
B033J18	B010F10	B028G03
B036N23	B015F11	B030G13
B012A02	B011F13	B026H09
B011A04	B011F14	B025H10
B015A06	B014F17	B026H11
B014A12	B015G01	B029I08
B010A20	B011G08	B027I11
B010A22	B012G08	B029I13

B009A08	B010G21	B031J21
B011B02	B016G23	B029K11
B009B04	B009G24	B028L15
B013B05	B014H01	B030L16
B014B07	B016H07	B027J18
B013B08	B009H18	B025J20
B010B11	B009H23	B031K02
B016B13	B001H24	B026L06
B013B17	B014H24	B030L19
B010B18	B009I03	B027L23
B010B19	B014I03	B026M18
B013B23	B009I04	B031M21
B010C04	B014I08	B026N09
B014C07	B011I19	B027N16
B015C08	B014J19	B028N16
B014C12	B011K04	B028N17
B016C01	B010K06	B028N24
B014D03	B016K12	B032N24
B012D04	B015L02	B032O06
B013D05	B012L03	B031O18
B016D12	B016L04	B032O19
B009D23	B012M18	B028P08
B014E02	B015N01	B030P23
B011E03	B016N02	B026M03
B011E09	B016N03	B031M04
B011E13	B015N04	B032M13
B011E15	B015N05	B028N11
B014F03	B016N09	B030N16
B009F05	B010N10	B026N17
B013F10	B013N10	B026N20
B011F13	B016N17	B032N22
B010F14	B012O02	B031O03
B015G01	B014O03	B031P14
B014G08	B016O08	B029P22
B012G17	B012O10	B044A20
B014H01	B012O13	B042B01
B016H07	B014O21	B046B06
B016H08	B015O21	B042B14
B011H24	B016P03	B043C01
B013I02	B014P05	B048C09
B011I19	B009P09	B046C14
B016I24	B009P10	B048C19
B014J03	B009P14	B043D02
B014J07	B013P20	B048D03
B010J08	B015P21	B042D07
B011J09	B011P22	B046D10
B011J11	B012P22	B047D11
B013J12	B010A23	B046D12
B013J14	B012C11	B041D13
B010J15	B013C11	B044A04
B014J19	B016D19	B044A17
B014K02	B016H05	B041B06

B012K04	B016H07	B041C14
B010K06	B012I04	B041D22
B011K07	B011J07	B045G05
B016K09	B011M08	B043H04
B016K12	B015M09	B042H11
B013K14	B009N05	B044E04
B010K19	B012N06	B044E07
B015L02	B014O06	B043E08
B014L07	B014O06	B047E09
B010L09	B015O18	B045F01
B016L12	B015P03	B045G13
B011L13	B010P22	B041H07
B011L15	B055A18	B044H09
B010L20	B053A21	B044H10
B012L23	B052B18	B044H11
B009M06	B056B18	B047H24
B009M09	B054B23	B043J11
B009M10	B050C11	B043K11
B015M11	B056C12	B044K11
B009M21	B055D12	B047K12
B015N01	B054D13	B041L15
B016N09	B050M16	B041L16
B013N10	B050M17	B041L17
B010N13	B052O14	B043I11
B015N14	B055O22	B045I15
B016N15	B056O21	B041I17
B014N16	B049O21	B048I17
B009N21	B050O21	B041I22
B014O12	B051O21	B047K19
B012O13	B052O21	B041N03
B013O15	B053O21	B041N12
B011O19	B054O20	B047N12
B014O21	B055O20	B041P04
B016P03	B052P24	B042P15
B014P05	B049P22	B041M09
B009P14	B056P22	B045M18
B012P22	B052P20	B047N07
B013P24	B051P19	B048N09
B027H03	B052P17	B046N21
B016H06	B066A20	B042O08
B013H09	B071A21	B048P02
B009H10	B071B14	B048P07
B013H13	B068B10	B043P22
B014H15	B068C15	B066C10
B014I06	B072D21	B070C15
B011I08	B065D23	B067C22
B015I12	B071D23	B069D19
B015K10	B068E19	B072D21
B012M18	B066E22	B067D23
B016N02	B068E22	B065E24
B016N03	B066F07	B071F11
B015N05	B068F07	B070G19

B011N06	B068G08	B068H07
B016N17	B066G08	B066H13
B012O02	B069G19	B071I06
B012O10	B065H08	B066I18
B016O16	B065H09	B067I23
B009P08	B067I01	B070J04
B009P09	B068I06	B069K22
B016P10	B067I11	B072L07
B009P13	B066I18	B065L23
B031A01	B065I19	B065L24
B027A03	B071I19	B065M07
B026A12	B066J10	B069N11
B025A13	B071J10	B067O07
B031A15	B067J15	B071O22
B027B01	B068J22	B069P23
B028B02	B069K10	B071P24
B026B07	B068L03	B082A03
B026B10	B070L07	B085A24
B025B12	B067L08	B082B03
B026B13	B065L20	B084C05
B030B15	B072L20	B086C05
B031B18	B068L23	B087D10
B029C03	B068L24	B083D13
B031C06	B069M01	B082E07
B028C07	B068M03	B082E19
B029C14	B068M08	B088F10
B025C15	B072M09	B084F24
B029C16	B069M18	B082H05
B028C17	B070N23	B082H08
B032C20	B068O14	B086I11
B028C21	B069O21	B086J16
B025C24	B102I11	B081K08
B025D06	B102K04	B083L03
B026D09	B103N13	B081L06
B027D12	B103N21	B084L08
B025D21	B098O14	B084M16
B029D23	B103O15	B088N09
B026E01	B100O20	B084O04
B032E02	B103O20	B083P12
B030E07	B082A03	B050A16
B025E10	B084A03	B050B08
B030E13	B087A03	B049B10
B026E14	B088A15	B054C06
B031E20	B071B21	B052D20
B030E21	B081B20	B051E18
B028F04	B087B20	B049F07
B027F08	B083C02	B054H13
B026F16	B085C05	B052L10
B026F17	B083C08	B054M08
B026F22	B088C22	B050M13
B028F23	B081C23	B052M17
B031G05	B084C23	B056N21

B032G21	B086D02	B052P15
B030G24	B087D11	B060C08
B028H01	B082D12	B064F20
B027H09	B083D12	B059G23
B031H10	B084D13	B061H10
B026H11	B082D19	B057H18
B029H12	B084E10	B064I06
B028H13	B088E12	B059K04
B032H18	B081E13	B061K11
B025H22	B087E13	B062K15
B029I01	B088E13	B062N13
B031I03	B082E18	B062O23
B032I05	B087E18	B080A08
B027I12	B087F04	B080A12
B026I13	B082F08	B074B02
B028I22	B084F10	B076B21
B025J01	B086F10	B078C04
B025J02	B087F10	B080C11
B032J04	B081F13	B078D12
B026K01	B082F13	B077D13
B031K02	B084F13	B077G09
B026K12	B082F20	B076I03
B028L01	B083G05	B075I13
B032L02	B088G08	B075I21
B026L06	B082G13	B080L04
B032L07	B086G13	B080L19
B029L10	B087G15	B073M04
B027L11	B082G17	B077M05
B030L19	B081G23	B079M06
B027L20	B087G23	B076M07
B027L23	B085H06	B079M07
B025M01	B084H09	B078M08
B032M03	B088H09	B074M11
B031M08	B082H12	B080O01
B029M11	B083H12	B074O06
B028M17	B082H13	B074P09
B026M18	B086H13	B073P11
B031M21	B088H15	B078P16
B025N01	B085H19	B093A08
B028N16	B086I01	B092B07
B028N17	B081I02	B093B18
B025N18	B082I02	B093C12
B028O04	B086I03	B096C11
B032O06	B087I03	B090C17
B030O13	B087I04	B096E13
B031O18	B086I05	B094F05
B032O19	B082I10	B094F06
B029O20	B083I10	B095F17
B030P23	B087I10	B089G18
B029A04	B081I12	B096H03
B028A14	B083I18	B092J04
B025B03	B084I18	B090L01

B025B05	B083I23	B095M21
B027B08	B084I23	B091O15
B030B14	B085J10	B095P14
B025B24	B087J10	B096P13
B028C02	B084J11	B103A07
B027C10	B082J12	B098A12
B027D07	B084J15	B099B08
B026D11	B088J17	B103C06
B032F02	B086J19	B100H03
B030F06	B081J24	B103J16
B027F12	B083K08	B101L03
B028F18	B084K08	B100N06
B029G02	B086K11	B103N24
B025G06	B082K12	B102P06
B026G14	B086K12	B113A04
B030H03	B088K13	B116A22
B031H14	B086K18	B113B01
B030H15	B082L03	B117B03
B025I09	B084L03	B120B13
B025I10	B083L04	B117C18
B031I17	B088L05	B119C24
B029I19	B083L09	B115E10
B031I21	B084L09	B116E15
B028J08	B081L17	B119F20
B027J18	B088L17	B120G01
B027J21	B088L18	B115G05
B032K08	B087M10	B118G10
B030K09	B086M13	B113H20
B026K10	B086M14	B120J17
B025K13	B082M15	B113L09
B031K18	B082M16	B120L16
B029K20	B082M17	B114M05
B028K23	B084M21	B118M11
B032L15	B087N03	B119M12
B028L18	B083N09	B118M15
B029L21	B082N11	B115M16
B027M09	B087N12	B115M22
B029M10	B082N17	B117N11
B029M14	B082N18	B117N12
B031N07	B084N18	B116O19
B028N09	B084N19	B113P09
B026N14	B085N19	B116P13
B028N21	B082O09	B111A04
B029O11	B084P08	B105B20
B031O12	B081P11	B110E01
B029P01	B081P21	B105E19
B027P04	B031A01	B107H13
B030P05	B026A12	B106I01
B026P11	B027B02	B109J10
B029P13	B025B03	B108K01
B027P16	B026B07	B111K13
B027P17	B025B12	B112L10

B031P19	B031B18	B107M05
B028P20	B025B21	B110M18
B055A01	B029C14	B111N11
B054A05	B025C15	B109O21
B056A08	B029C16	B125A15
B056A11	B032C20	B128A17
B055A15	B026C23	B127B08
B049A16	B025C24	B122B18
B053A18	B026D03	B125C11
B056A19	B026D09	B121G05
B049A21	B029D23	B123J03
B056A24	B029E01	B122K07
B055B03	B032E02	B127K12
B054B07	B025E10	B122L08
B049B14	B031E20	B122L19
B052B16	B030E21	B123M07
B053B19	B030E22	B128M17
B052B24	B031F02	B125N23
B054C02	B032F02	B121O09
B054C05	B026F16	B127O19
B054C07	B026F22	B126P18
B052C08	B028F23	B121P21
B050C11	B029G02	B138B05
B049C12	B026G05	B143B11
B055C20	B025G06	B142C16
B050C22	B027G08	B144D05
B056C23	B026G14	B141D06
B054D02	B032G16	B141D15
B050D10	B032G21	B139G21
B054D16	B028G23	B144H10
B054D19	B028H01	B142H17
B050D20	B030H15	B143H19
B051E03	B031H18	B137J06
B054E06	B031I03	B143J21
B056E07	B032I05	B144K20
B050E08	B031I17	B142M04
B054E09	B029I19	B140M06
B051E16	B028I22	B138N12
B056E21	B025J01	B137O06
B049E22	B025J02	B144O07
B052F08	B028J08	B141P12
B055F16	B031J15	B139P19
B049G07	B027J18	B150B18
B051G17	B025J20	B151B20
B050H02	B027J21	B145C08
B054H03	B027J22	B147C09
B050H04	B031K14	B146C12
B049H05	B031K18	B146C13
B051H08	B029K20	B147D01
B054H20	B028K23	B150D02
B052H23	B025L15	B148E04
B053I05	B032L15	B151E17

B053i16	B030L19	B152E17
B052I21	B030L22	B151F04
B052I24	B027L23	B145F16
B049J01	B029M10	B152G20
B051J12	B029M11	B151I22
B053J16	B029M14	B150J11
B055J21	B026M18	B148K04
B051j24	B028M20	B152L03
B054K05	B031M21	B145L11
B054K06	B032M24	B150L24
B056K09	B027N16	B151M09
B049K11	B028N16	B146M17
B056K12	B028N17	B146P04
B050K21	B025N18	B131A13
B055L06	B032N20	B135A14
B049L09	B028N21	B136A16
B049L10	B029O11	B131A18
B049L12	B030N15	B130B17
B056M03	B031O18	B136C01
B050M04	B032O19	B134C21
B050M07	B029O20	B129D01
B052M08	B027O23	B135D19
B052M09	B029P01	B130D21
B056M11	B029P13	B136E02
B054M14	B030P20	B129E12
B053M23	B032P21	B132G09
B051N02	B030P23	B132G11
B056N06	B028A02	B136G11
B050N08	B031A03	B130G19
B050N10	B031A04	B130G23
B053N11	B027A08	B132H07
B055O01	B028A11	B136H13
B049O06	B025B07	B129H18
B051O07	B030B08	B131I09
B051O10	B030B10	B129I14
B053O12	B028C04	B133I16
B053O13	B028E01	B129J10
B051O14	B032H04	B131J14
B050O16	B028H12	B132K09
B051O17	B025J04	B135K20
B051O19	B028L05	B133L02
B050O21	B028M05	B133M06
B049P07	B046B12	B133M07
B050P08	B046B13	B135M17
B053P13	B041C12	B129N09
B053P16	B044C12	B132N11
B050P17	B047D01	B129N12
B056P19	B042D02	B131N17
B041A06	B044D05	B131O20
B044A17	B042D12	B134P18
B041B02	B047D17	B164A07
B044B03	B041F03	B166A15

B041B06	B047J14	B165A19
B045B14	B043J15	B163A22
B043B16	B044A04	B161C07
B048C03	B046A05	B163C15
B041C05	B041A06	B164D06
B044C08	B043A16	B163F09
B041C11	B044A17	B162G09
B046C12	B042A19	B163G20
B043D11	B047A20	B168H01
B041E01	B041B06	B168I22
B046E06	B045B11	B167L12
B045E07	B043B15	B165M01
B043E13	B043B16	B181A10
B046F04	B047B23	B177A11
B041F05	B048C07	B181D02
B042F06	B041C14	B181F13
B048F09	B042C18	B182G10
B047F16	B047C18	B184H09
B046F17	B043C19	B178J10
B048G08	B042C20	B183J11
B048G13	B045C23	B179L05
B041G14	B047C23	B180M02
B043H13	B042D02	B178M16
B045I01	B048D04	B182M18
B043P02	B043D11	B177N22
B084A01	B046D13	B180O09
B087A02	B045E09	B178O12
B086A08	B047E16	B184P16
B086A11	B041E18	B184P17
B088A15	B042E19	B185A14
B087A16	B042E20	B186A16
B088A18	B041E21	B185D03
B086A20	B042F06	B189E01
B084A22	B047F16	B188E22
B083B02	B046F17	B188E24
B081B03	B042G01	B188F09
B081B04	B048G02	B189F20
B088B05	B045G05	B188H08
B083B06	B042G06	B186H19
B081B10	B043H04	B188H21
B086B11	B043H05	B190J10
B082B12	B042H11	B187J13
B083B13	B041H15	B186O02
B087B14	B048H21	B192P09
B088B16	B048H23	B192P14
B086B22	B042I02	B174A15
B087B23	B047I05	B174D12
B081B24	B043I11	B170E16
B083C03	B041I17	B170H19
B087C04	B048I17	B170H22
B083C05	B041I18	B176J04
B088C06	B042I21	B171J08

B086C07	B047I21	B173J11
B086C08	B041I22	B176K10
B082C13	B048J02	B176P07
B084C16	B043J07	B176P09
B086C17	B042J13	B159C14
B081C20	B045J15	B159C18
B085C22	B043J16	B158D18
B081D02	B041J21	
B087D04	B043J22	
B081D07	B047K13	
B083D08	B045K16	
B087D09	B047K19	
B082D10	B043L02	
B082D10	B047L03	
B082D12	B045L10	
B087D15	B045L17	
B085D16	B042L19	
B087D21	B043L20	
B087D22	B044L20	
B086E03	B047M01	
B084E07	B043M03	
B086E08	B048M14	
B087E09	B041M23	
B086E11	B044N01	
B087E12	B041N03	
B086E13	B045N05	
B085E17	B047N06	
B081E19	B041N12	
B085E20	B046N12	
B088E21	B047N17	
B085E22	B047N18	
B088E23	B043O03	
B082E24	B041O06	
B083F02	B046O09	
B086F04	B041P04	
B088F05	B047P10	
B088F06	B041P11	
B085F07	B041P12	
B084F08	B042P15	
B083F10	B041P16	
B081F12	B044P21	
B082F15	B117A03	
B081F17	B116A04	
B085F20	B116A06	
B085F21	B113A08	
B083F24	B113A13	
B086G01	B115A13	
B081G02	B120A13	
B081G03	B120B05	
B082G05	B115B16	
B081G11	B115B20	
B084G12	B116B20	

B088G16	B115B19
B083G21	B117B23
B085G22	B113C11
B083H02	B117C15
B081H03	B114C14
B083H05	B118C20
B083H06	B115D20
B088H08	B116D20
B084H11	B117D24
B087H13	B117E09
B084H16	B120E19
B088H17	B115E21
B084H18	B119F17
B083H22	B120G21
B083H24	B120H12
B087I02	B116H13
B088I03	B115H16
B081I16	B114H14
B084I17	B113I10
B088I18	B117I11
B081I21	B120I11
B088I22	B117I12
B083I23	B120I12
B069A01	B115I13
B069A05	B113I14
B069A06	B118I16
B070A12	B117I18
B066A14	B114I19
B065A15	B117I19
B066A21	B113I20
B068A23	B120I20
B065B13	B118I21
B065B18	B119J08
B071B21	B117J11
B065B22	B113J12
B066C02	B115J12
B070C03	B119J15
B066C07	B119J18
B070C10	B115J19
B067C11	B120J21
B068C13	B115K07
B070C14	B119K08
B069C15	B120K09
B067C16	B120K10
B065C17	B116K18
B066C22	B118K20
B066D02	B118L07
B066D06	B113L12
B066D08	B116L12
B065D10	B120L12
B072D13	B118L16
B070D20	B119M03

B066D21	B114M06
B072D22	B116M06
B065E05	B113M18
B065E08	B116N21
B069E09	B117N21
B070E15	B115O01
B072E20	B113O06
B071F07	B119O20
B067F08	B114O21
B065F09	B117O21
B072F20	B119O21
B069G09	B119P01
B068G11	B114P06
B068G13	B119P06
B069G14	B113P12
B072G18	B113P13
B072G22	B117P16
B065G24	B119P17
B065H10	B135A01
B069H12	B132A02
B068H16	B136A02
B068H18	B134A06
B072H21	B131A12
B071H23	B129A20
B067H24	B134A23
B065I03	B129B02
B071I04	B133B02
B071I10	B136B15
B065I13	B135B16
B069I15	B134B18
B066I20	B130B19
B068I24	B133C06
B065J03	B129C09
B069J07	B136C09
B066J08	B134C17
B066J09	B129C20
B066J11	B132C20
B066J13	B134C22
B072J17	B135D17
B067J21	B132D20
B068J22	B129D21
B069J23	B133E02
B065J24	B130E09
B070K02	B130E13
B071K06	B130E16
B070K08	B132E22
B068K10	B133F16
B072K11	B134F20
B071K18	B135G02
B068L01	B130G13
B065L03	B132G15
B071L05	B130G17

B071L07	B131G17
B072L08	B132G17
B065L11	B136G19
B066L15	B129H13
B070L20	B135H14
B071L24	B135H18
B065M05	B130H20
B066M08	B130I13
B070M09	B131J07
B072M10	B132J07
B069M11	B130J11
B067M16	B132J12
B065M18	B136J17
B070N01	B130J19
B068N04	B133K03
B065N05	B130J04
B068N09	B132K04
B070N11	B129K09
B066N13	B136K09
B072N16	B131K11
B069N21	B132K11
B072O01	B136K15
B071O04	B129L09
B065O06	B136L09
B065O07	B130L10
B071O09	B136L12
B065O10	B132L13
B065O12	B135L13
B067O16	B129L16
B066O21	B132L17
B065P01	B132M12
B068P07	B136M12
B070P08	B130M21
B065P10	B133M23
B066P11	B132M24
B070P12	B131N04
B071P13	B134N05
B072P14	B135N09
B067P15	B130O06
B070P16	B132O06
B068P19	B132O10
B065P24	B133O14
B092A03	B134O17
B089A04	B134O18
B095A05	B134P09
B091A13	B135P10
B091A14	B136P13
B092A14	B132P17
B092A17	B133P19
B090A18	B132P20
B089A20	B064A15
B092A20	B057A18

B089B03	B057C11
B089B04	B057L07
B091B06	B063L03
B092B12	B057L11
B094B13	B061M13
B092C01	B059N13
B089C02	B063O07
B094C03	B061O10
B089C06	B057O14
B094C07	B061P12
B094C08	B064P12
B094C11	B061P20
B090C12	B078B07
B090C16	B073B08
B096C19	B079B08
B090C20	B077C07
B094C23	B074D02
B094D01	B079D02
B092D02	B075D04
B096D06	B075D07
B096D07	B073D10
B095D08	B077D10
B096D13	B075D11
B089D23	B076D11
B090E03	B077D13
B094E05	B077E08
B091E14	B078E09
B089E16	B079F10
B089E17	B074F11
B089E18	B079G02
B096E24	B080G02
B089F07	B073G10
B094F11	B079G11
B089F12	B080H05
B089F19	B073H06
B094F20	B076H07
B093F21	B079H07
B090G02	B080H08
B089G05	B078H09
B092G05	B079H09
B090G06	B080H10
B092G17	B073H06
B096G17	B080H06
B089H07	B077H08
B095H11	B076H10
B089H13	B076J02
B096H13	B078J06
B095H15	B078J08
B091H16	B079J08
B090H17	B076J09
B091H20	B080J09
B092H22	B073J10

B095I01	B076J10
B096I04	B074J11
B093H10	B075J14
B092H12	B073J16
B090H13	B077J16
B092H13	B074J18
B090H19	B078L11
B095J01	B074M03
B093J04	B080M07
B092J08	B077M09
B095J08	B073M16
B091J10	B077M17
B089J19	B074N06
B092J19	B077N06
B089K02	B073N12
B089K04	B080N12
B094K05	B078O09
B091K15	B074P01
B092K15	B073P02
B091K18	B073P09
B095L10	B080P09
B096L11	B074P12
B089L17	B080P24
B092L18	B096A07
B090L19	B090A08
B091M05	B094B18
B090M07	B094B21
B091M08	B093C14
B092M13	B095C21
B090N01	B093D05
B094N10	B089D06
B089N17	B095D06
B095N20	B092D07
B089N21	B094D14
B095O07	B095D18
B091O08	B096E02
B091O10	B089E03
B092O10	B096E13
B94O11	B092F03
B089O12	B089F07
B090P04	B089F09
B089P05	B096F09
B094P08	B092F14
B090P19	B091F14
B098A05	B096G02
B097A12	B095G05
B100A12	B089G08
B104A14	B095G08
B103B02	B089G14
B104B03	B092H02
B102B10	B090H13
B102B11	B093H13

B101B12	B090H15
B101B14	B092H18
B099B16	B094H20
B103C01	B093H23
B102C03	B095I07
B100C04	B091I16
B097C13	B091J01
B104C15	B092J01
B097D01	B089J04
B100E02	B096J04
B103F23	B093J12
B100H02	B094J13
B101I02	B095J20
B099L09	B089J21
B105M03	B096K02
B100N23	B094K09
B101O09	B092K18
B100O21	B095L07
B099O24	B096L07
B102P01	B091L11
B099P16	B091L12
B100P21	B089L17
B104P21	B096L18
B113A03	B089L19
B116A06	B091L19
B119A08	B089L20
B118A09	B096L20
B118A16	B089L21
B114A23	B095M05
B117B10	B089M06
B113B11	B091M06
B118B11	B096M06
B113B12	B089M08
B120B12	B095M08
B118B15	B096M08
B117B16	B093M09
B118B18	B089M13
B113C03	B093M13
B119C03	B092M16
B115C09	B090M17
B116C10	B095N03
B120C11	B096N04
B118C15	B089N05
B117C16	B092N05
B116C23	B089N06
B119C23	B094N06
B118D02	B089N07
B114D10	B090N07
B114D11	B094N02
B119D12	B095N07
B120D12	B096N08
B118D15	B092N11

B114D20	B096N17
B114D22	B091N15
B120E01	B093O06
B114E12	B090O07
B115E14	B093O07
B116E16	B094O07
B115E18	B092O09
B114E20	B090O10
B117F07	B094O10
B119F14	B091O11
B114F15	B093O12
B113G03	B092P21
B118G03	B105A04
B115G11	B112A09
B119G15	B105D05
B116G20	B105I03
B113G21	B109I08
B120H01	B110I13
B116H12	B108I16
B120H14	B107I17
B117H15	B106J02
B117H18	B108J04
B118H19	B111J04
B118H22	B111J17
B115H23	B112K01
B113I12	B108K07
B117I13	B108K09
B113I16	B112L02
B118I16	B109L08
B120I17	B110L07
B114I19	B112L12
B119I22	B109L23
B113J13	B107M11
B120J14	B105M13
B117J15	B106M14
B120J20	B112M14
B118J21	B106N03
B114J22	B109N03
B116J22	B109N06
B117J22	B106N12
B118K06	B110N12
B116K10	B111N13
B119K12	B108N14
B118K14	B110N18
B114K19	B107O14
B120K20	B108O14
B114L05	B109P12
B115L08	B107P14
B113L18	B112P21
B118L18	B121A18
B114L19	B125A18
B118L21	B124B12

B118M08	B128B12
B118M10	B125B14
B116M12	B123B18
B114M18	B124B18
B119N01	B022C03
B118N02	B127C04
B114N04	B123C10
B116N05	B128C11
B116N08	B125C22
B113N09	B121E18
B116N09	B124E19
B120N11	B127G02
B119N17	B128G08
B119O01	B127G10
B117O03	B123I03
B116O06	B121J07
B119O07	B127J07
B116O14	B128J07
B115O14	B123J08
B115O17	B122K02
B119O18	B127K03
B118O23	B128K04
B116P08	B123K06
B120P08	B122L03
B120P09	B127K10
B117P10	B121L12
B120P11	B124M08
B119P18	B121N05
B119P21	B128N05
B119P24	B128N06
B130A04	B121N07
B134A04	B124N07
B133A06	B127N07
B131A10	B122N18
B135A11	B124P02
B136A13	B121P13
B131A15	B128P13
B131A17	B124P12
B129A20	B128P12
B134A21	B123P14
B136B02	B127P16
B131B04	B141B05
B131B05	B141D11
B131B06	B144D10
B134B07	B143E13
B131B08	B142E14
B135B14	B140E16
B133B15	B139F13
B133B18	B141F13
B130B23	B143F16
B133C05	B137G11
B136C10	B140H03

B135C11	B143H08
B131C12	B143H09
B134C16	B144H09
B129C24	B139H10
B129D01	B140H13
B129D04	B139H17
B134D07	B140H18
B132D08	B141J08
B135D11	B138J10
B129E01	B139J10
B130E04	B137J17
B134E09	B139J17
B135E10	B141J17
B133E14	B142K08
B133E15	B144K15
B129E16	B138K13
B134E18	B143K13
B136E19	B140L05
B129E21	B147L05
B136E24	B141L08
B132F02	B141L09
B133F04	B137L15
B135F06	B137L16
B130F07	B139L16
B136F08	B140L20
B130F09	B139L20
B136F09	B142M03
B135F10	B144M06
B129F11	B138M07
B132F12	B139M07
B129F16	B140M08
B135F16	B137M17
B135G03	B144M17
B136G08	B143M21
B133G09	B140N08
B133G10	B141N08
B132G13	B141N15
B131G17	B141N17
B134H05	B139O11
B129H11	B143O16
B133H12	B138P05
B136H22	B042P08
B129I01	B139P20
B134I02	B153A06
B131I03	B156A08
B131I05	B157C07
B132I12	B153H07
B129I13	B157L06
B131I16	B155M15
B133J03	B159N17
B133J05	B157P02
B134J06	B175J08

B135J07	B175J09
B136J19	B171L10
B131J21	B175N08
B130K04	B175N10
B132K06	B170N12
B131K08	B171N12
B135K09	B172N12
B133K10	B172O01
B134K15	B188B08
B129K17	B188A13
B129K20	B191F11
B134K22	B191G13
B130L02	B185H10
B130L03	
B132L05	
B133L05	
B133L06	
B131L07	
B131L09	
B131L11	
B131L15	
B134L24	
B129M06	
B135M09	
B134M10	
B130M11	
B132M12	
B134M12	
B134M16	
B129M17	
B136M19	
B133M21	
B135M22	
B129M23	
B136N03	
B130N04	
B130N10	
B133N10	
B132N11	
B135N12	
B133O09	
B130O14	
B136O22	
B129P11	
B129P14	
B111A05	
B107A06	
B108A09	
B111A08	
B112A11	
B108A14	
B109A14	

B108A15
B106A16
B106A19
B109B04
B111B05
B105B10
B110B11
B108B12
B109B14
B112B17
B112B20
B107C03
B108C06
B109C06
B106C10
B110C13
B106C14
B106C15
B108C15
B110C16
B112C17
B112C20
B109C21
B109D01
B106D02
B112D06
B107D08
B109D09
B108D13
B112D17
B105D19
B106E01
B106E02
B107E08
B109E09
B108E13
B112E17
B105E19
B110F04
B108F05
B106F07
B111F08
B105F12
B112F12
B107F13
B112F16
B108F21
B105F23
B111G07
B112G09
B108G13
B108G14

B108G15
B110G17
B109G18
B111H04
B106H05
B111H06
B105H07
B105H08
B110H08
B111H09
B111H11
B107H13
B108H17
B105H24
B112I01
B106I03
B106I04
B111I04
B108I02
B109I02
B109I05
B111I08
B110I13
B106I15
B109I16
B108I17
B111I18
B111I19
B105J03
B111J03
B111J04
B111J06
B106J07
B107J08
B111J10
B106J11
B106J15
B112J16
B107J17
B110J18
B111J24
B108K03
B110K04
B106K06
B105K07
B110K07
B109K10
B110K11
B106K13
B112K16
B106K18
B108L02

B112L05
B106L06
B111L06
B109L09
B112L13
B105L15
B111L18
B112L24
B110M02
B109M03
B105M04
B106M05
B110M10
B111M11
B107M18
B108M18
B112M19
B112M22
B108M24
B111N07
B110N03
B105N04
B110N05
B107N09
B106N10
B107N10
B110N11
B107N18
B107N21
B107O04
B106O09
B105O10
B108O14
B111O14
B106O24
B111P01
B105P02
B108P02
B110P03
B112P03
B112P04
B105P06
B108P06
B109P11
B111P12
B112P13
B108P14
B111P17
B110P18
B107P18
B108P18
B105P19

B111P19
B108P20
B106P22
B107P22
B138A08
B142A08
B141A14
B143A18
B137A16
B141A18
B139A21
B143B07
B140B08
B142B13
B144B13
B141B15
B142B16
B141C04
B137C08
B139C09
B139C10
B143C13
B144C15
B144C16
B142C17
B141C18
B144C20
B139C21
B139D05
B141D05
B139D10
B137D11
B141D11
B144D13
B143D18
B143D19
B141E03
B142E10
B144E11
B137E15
B144E17
B141F04
B141F05
B142F06
B142F08
B138F10
B143F10
B139F12
B143F19
B144G06
B143G09
B140G16

B137G21
B140H04
B144H05
B138H07
B142H08
B137H15
B143I06
B141I08
B141I09
B144I15
B144J04
B139J05
B141J05
B142J11
B140J21
B137K04
B142K06
B144K09
B139K11
B138K12
B141L08
B142L11
B140L14
B138L15
B139L16
B138L17
B142L18
B138L24
B140M02
B137M05
B144M05
B138M06
B143M13
B137M18
B141M18
B143M22
B138M24
B144N04
B137N05
B144N05
B139N08
B143N12
B139N13
B143N15
B141N17
B141N18
B143O07
B137O02
B140O09
B138O10
B137O12
B144O12

B143O19
B137O21
B140O21
B142P07
B137P08
B137P20
B140P20
B121A02
B128A02
B121A04
B127A06
B128A07
B123A09
B124A09
B124A11
B126A11
B126A12
B128A14
B123A18
B125A18
B121B01
B125B01
B123B03
B124B03
B127B05
B128B08
B125B10
B127B11
B126B12
B126B13
B122B15
B126B15
B125B16
B128B19
B128C03
B127C04
B127C06
B125C08
B121C09
B127C09
B123C11
B124C13
B125C16
B127C16
B123C18
B121D02
B124D02
B125D03
B126D05
B123D06
B122D08
B126D20

B125E01
B122E03
B127E04
B128E08
B128E10
B128F02
B124F04
B125F09
B121F10
B128F10
B123F11
B121G02
B125G07
B127G11
B128H02
B121H04
B126H05
B123H06
B124H08
B128H09
B123H11
B128H11
B121I02
B125I02
B128I02
B123I03
B128I11
B127I13
B126I15
B127I15
B121I01
B121J01
B123J02
B125J03
B128J04
B121J06
B128J07
B122J09
B125J10
B127J13
B124J16
B127J17
B121J24
B127K03
B126K04
B127K11
B126K12
B122L02
B127L02
B124L03
B124L05
B126L07

B122L09
B123L11
B124L11
B125L12
B124M03
B121M04
B121M05
B121M06
B128M06
B122M07
B128M07
B123M09
B125M09
B121M11
B121N03
B123N04
B123N06
B122N09
B124N09
B128N09
B128N11
B128N13
B128N14
B125N16
B124N17
B121N21
B128O02
B125O06
B122O06
B121O09
B124O09
B124O10
B122O16
B126O17
B127O17
B123O18
B125O18
B128O21
B125P03
B124P08
B122P10
B124P14
B122P15
B123P16
B122P21
B128P21
B161A02
B162A12
B163A14
B164A15
B168A15
B161A21

B168A21
B162A22
B164A22
B164A24
B165B02
B165B08
B167B14
B167B16
B168B19
B162B18
B162B21
B164B21
B163B22
B167C03
B168C05
B168C07
B166C08
B164C09
B161C16
B164C18
B163C22
B164C22
B167D05
B167D08
B162D24
B168D24
B164E10
B162E16
B163E16
B164E18
B167F03
B165F04
B168F07
B165F09
B161F10
B167F11
B161F14
B162F17
B167F19
B163F21
B167G01
B168G03
B166G09
B162G10
B163G15
B166G17
B165G19
B163G24
B166H01
B164H02
B164H07
B166H15

B163H18
B162H20
B161H22
B166H22
B168I03
B162I04
B162I05
B167I22
B165J01
B168J02
B161J04
B161J06
B168J11
B163J14
B168J11
B163J14
B166K02
B165K16
B161K22
B163L01
B165L02
B164L03
B167L03
B165L04
B163L11
B167L16
B162L17
B164L18
B165L19
B163L20
B163M03
B167M07
B163M08
B164M18
B163M21
B168M21
B164M23
B164N06
B167N06
B167N08
B166N20
B168O11
B164O20
B167P07
B161P08
B168P08
B164P09
B166P10
B164P13
B162P21
B162P22
B149A09

B151A10
B149A11
B145B05
B145B07
B145B08
B152B08
B149B14
B149B16
B145B20
B146C14
B147C14
B146C15
B145C18
B152C18
B145C19
B151C20
B151C23
B150C24
B145D02
B152D03
B146D04
B151D05
B147D06
B145D07
B147D11
B148D11
B150D17
B148D21
B146D23
B151D23
B147E01
B149E16
B148E19
B147E20
B152F04
B151F10
B147F12
B146F13
B150F14
B146G15
B145H04
B145H05
B152H08
B147H10
B149H11
B151H18
B145H20
B148H23
B151I08
B149I09
B150I12
B151I21

B147I23
B150J04
B148J06
B146J09
B147J09
B148J09
B147J12
B148J14
B151J16
B145J19
B152J19
B148J21
B147J23
B146K08
B146K09
B149K10
B145K12
B152K12
B150K14
B147K15
B152L04
B149L07
B145L14
B150L16
B151L18
B150L23
B151M03
B146M05
B146M06
B149M06
B147M07
B151M07
B145M10
B152M11
B151M23
B152N05
B148N06
B146N10
B149N11
B147N15
B150N21
B152N23
B151O01
B146O05
B151O07
B147O09
B150O09
B145O10
B150O10
B147O16
B146O18
B149P01

B146P02
B148P04
B149P07
B149P08
B150P09
B147P12
B151P12
B147P14
B147P16
B151P20
B152P21
B147P22
B145P23
B148P23
B151P24
B145P19
B153A15
B153A19
B156B08
B158B09
B155B10
B153B18
B154B20
B153B24
B160C03
B160C04
B157C12
B155C13
B158C14
B156C15
B160C15
B154C20
B153C24
B154D06
B160D13
B156D17
B153E05
B160E20
B153F05
B160F05
B153F06
B154F07
B155F09
B154F13
B156G08
B159G10
B154H03
B154H08
B158H08
B153H11
B156H11
B155H18

B160H23
B160I07
B158I12
B158J04
B160J07
B157J08
B157K02
B155K03
B157K06
B157K09
B156K14
B158K15
B159K15
B154K19
B153L03
B157L04
B160L05
B153L07
B154L12
B153L13
B156L13
B153L14
B157L16
B158L18
B153M05
B157M05
B154N12
B157N15
B154N03
B154O01
B160O09
B159O10
B159O16
B153P01
B156P01
B153P03
B157P05
B179A09
B184A10
B182A15
B177B06
B180B07
B179B08
B180B08
B178B20
B177B06
B180B07
B180B08
B179B08
B178B20
B180C03
B184C04

B180C07
B180C12
B179C13
B184C22
B177D03
B178D09
B181D09
B181D11
B182D11
B180D12
B180D15
B179D19
B182D20
B183E04
B180E09
B183E11
B180F09
B184F10
B179F10
B179F11
B183F11
B183G03
B177G13
B181H04
B179H05
B179H06
B182H07
B177I03
B183I06
B181I07
B178I13
B182I14
B183J02
B177J08
B181J08
B181J12
B183J14
B183K08
B179L03
B183L08
B184L24
B179M03
B180M03
B183M06
B180M07
B181M09
B177N04
B180N06
B178N08
B183N08
B179N09
B178N10

B181N16
B183O03
B182O21
B180P06
B184P06
B182P12
B191A05
B186A14
B191A19
B192A20
B188B01
B189B02
B190B04
B192B08
B187B11
B191B11
B188B12
B188B16
B186B17
B186B24
B189C04
B187C05
B192C11
B190C20
B190D07
B188D10
B190D13
B192D15
B185D18
B188D19
B189D20
B192D21
B188D22
B191E13
B191E16
B188E18
B192E20
B186E21
B186F03
B185F10
B185F13
B192F14
B190F15
B192F15
B185F18
B188F21
B188G06
B190G09
B190G10
B185G11
B188G13
B191G19

B192H10
B192H11
B192H12
B192H13
B185I08
B186I01
B191I13
B186I20
B185J06
B190J07
B189J08
B190J11
B186J17
B185J19
B186J20
B191K09
B187K15
B185K18
B192L11
B190M11
B189N05
B192N07
B188O08
B189O08
B185O16
B191O18
B191O20
B186P09
B185P21
B191P21
B172A02
B176A05
B169A08
B172A13
B169A16
B172A16
B175B03
B170B13
B174B20
B175C03
B171D06
B172D12
B172E01
B175E06
B170E15
B170E13
B173E18
B170E19
B170F02
B175F08
B170F11
B172F11

B169F16
B169F17
B172F17
B176F19
B169F21
B174G04
B169G19
B175H01
B169H09
B172H15
B172H18
B175I15
B171I18
B169J15
B175J16
B170J17
B169J19
B169K18
B169L03
B175L12
B170L14
B175L14
B172L18
B175N09
B172N13
B175O02
B175P12
B169P14
B176P15
B169P18
